# A meta-analysis of epigenome-wide association studies of ultra-processed food consumption with DNA methylation in European children

**DOI:** 10.1186/s13148-024-01782-z

**Published:** 2025-01-07

**Authors:** Joana Llauradó-Pont, Nikos Stratakis, Giovanni Fiorito, Evangelos Handakas, Alexander Neumann, Henrique Barros, Anne Lise Brantsæter, Kiara Chang, Leda Chatzi, Janine F. Felix, Regina Grazuleviciene, Vincent W. V. Jaddoe, Marianna Karachaliou, Marion Lecorguillé, Carla Lopes, Christopher Millett, Rosemary R. C. McEachan, Eleni Papadopoulou, Remy Slama, Eszter P. Vamos, Paolo Vineis, Martine Vrijheid, John Wright, Trudy Voortman, Mariona Bustamante, Oliver Robinson, Camille Lassale

**Affiliations:** 1 ISGlobal, Barcelona, Spain; 2 Clinical Bioinformatics Unit, IRCCS Instituto Giannina Gaslini, Genova, Italy; 3 Medical Research Council Centre for Environment and Health, School of Public Health, Imperial College London, London, UK; 4 Department of Child and Adolescent Psychiatry/Psychology, Erasmus MC, University Medical Center Rotterdam, Rotterdam, The Netherlands; 5 Generation XXI Study Group, EPIUNIT/ITR- Laboratory for Integrative and Translational Research in Population Health, Institute of Public Health, University of Porto, Porto, Portugal; 6 Department of Food Safety, Centre for Sustainable Diets, Norwegian Institute of Public Health, Oslo, Norway; 7 Public Health Policy Evaluation Unit, School of Public Health, Imperial College London, London, UK; 8 Department of Preventive Medicine, University of Southern California, Los Angeles, USA; 9 Generation R Study Group, Erasmus MC, University Medical Center Rotterdam, Rotterdam, The Netherlands; 10 Department of Pediatrics, Erasmus MC, University Medical Center Rotterdam, Rotterdam, The Netherlands; 11 Department of Environmental Sciences, Vytautas Magnus University, Kaunas, Lithuania; 12 Université Paris Cité and Université Sorbonne Paris Nord, Inserm, INRAE, Center for Research in Epidemiology and Statistics (CRESS), Paris, France; 13 NOVA National School of Public Health, Public Health Research Centre, Comprehensive Health Research Center, CHRC, NOVA University Lisbon, Lisbon, Portugal; 14 Division of Health Service, Global Health Cluster, Norwegian Institute of Public Health, Oslo, Norway; 15 Team of Environmental Epidemiology, IAB, Institute for Advanced Biosciences, Inserm, CNRS, CHU-Grenoble-Alpes, University Grenoble-Alpes, CNRS, Grenoble, France; 16 Universitat Pompeu Fabra (UPF), Barcelona, Spain; 17 Consortium for Research on Epidemiology and Public Health (CIBERESP), Instituto de Salud Carlos III, Madrid, Spain; 18 Bradford Institute for Health Research, Bradford Teaching Hospitals NHS Foundation Trust, Bradford, West Yorkshire UK; 19 Department of Epidemiology, Erasmus MC, University Medical Center Rotterdam, Rotterdam, The Netherlands; 20 Consortium for Biomedical Research - Pathophysiology of Obesity and Nutrition (CIBEROBN), Instituto de Salud Carlos III, Madrid, Spain

**Keywords:** Epigenome-wide association study (EWAS), Ultra-processed food (UPF), DNA methylation, Children, Nutrition

## Abstract

**Background/objective:**

There is limited knowledge on how diet affects the epigenome of children. Ultra-processed food (UPF) consumption is emerging as an important factor impacting health, but mechanisms need to be uncovered. We therefore aimed to assess the association between UPF consumption and DNA methylation in children.

**Methods:**

We conducted a meta-analysis of epigenome-wide association studies (EWAS) from a total of 3152 children aged 5–11 years from four European studies (HELIX, Generation XXI, ALSPAC, and Generation R). UPF consumption was defined applying the Nova food classification system (group 4), and DNA methylation was measured in blood with Illumina Infinium Methylation arrays. Associations were estimated within each cohort using robust linear regression models, adjusting for relevant covariates, followed by a meta-analysis of the resulting EWAS estimates.

**Results:**

Although no CpG was significant at FDR level, we found suggestive associations (*p*-value < 10^–5^) between UPF consumption and methylation at seven CpG sites. Three of them, cg00339913 (PHYHIP), cg03041696 (intergenic), and cg03999434 (intergenic), were negatively associated, whereas the other four, cg14665028 (NHEJ1), cg18968409 (intergenic), cg24730307 (intergenic), and cg09709951 (ATF7), were positively associated with UPF intake. These CpGs have been previously associated with health outcomes such as carcinomas, and the related genes are mainly involved in pathways related to thyroid hormones and liver function.

**Conclusion:**

We only found suggestive changes in methylation at 7 CpGs associated with UPF intake in a large EWAS among children: although this shows a potential impact of UPF intake on DNAm, this might not be a key mechanism underlying the health effects of UPFs in children. There is a need for more detailed dietary assessment in children studies and of intervention studies to assess potential epigenetic changes linked to a reduction in UPF in the diet.

**Graphical abstract:**

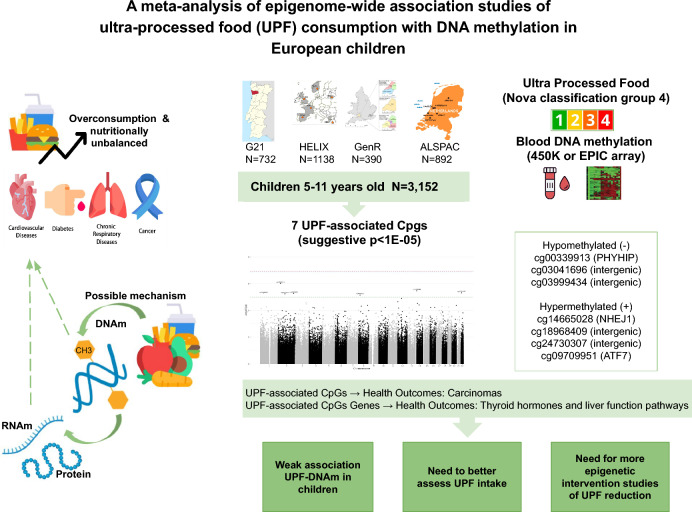

**Supplementary Information:**

The online version contains supplementary material available at 10.1186/s13148-024-01782-z.

## Introduction

Ultra-processed foods (UPFs) are industrially formulated products that undergo a series of physical, chemical, and biological processing. UPFs often lack intact healthy food components, contain various additives, and are energy dense and nutritionally poor (i.e. high in free sugar, salt, and saturated and trans fats but low in dietary fibre, and micronutrients) [1] and usually have softer textures [2]. Their consumption has risen dramatically in the last decades among countries worldwide. UPF intake (percentage of total calories consumed) in the adult population ranges from 15 to 57% in Romania and in the UK, respectively [3]. In school-aged children, it was estimated that UPF comprised 65.4% of daily calorie intake in a representative sample in the UK (4–10 years) [4], 66% in US children [5], while it was lower in Belgian children aged 3–9 years (33.3%) [6] and in Colombia (19%) [7].

The consumption of UPF has been associated with a range of adverse health outcomes in adults, as shown in a recent umbrella review [8]. In children, UPF intake has also been associated with an increase in energy intake and weight gain [9] and the modulation of biological pathways related to adiposity accumulation [10, 11], increasing risk of obesity-related diseases [12, 13], and insulin resistance [14]. The rise in obesity prevalence in children [15] is a significant public health concern with far-reaching ramifications, as childhood obesity tracks into adolescence and adulthood [16]. Obesity is associated with an elevated risk of the development of non-communicable diseases (NCDs) [17], in particular cardiometabolic diseases, type 2 diabetes, and some cancers [18].

Epigenetic mechanisms have been implicated in the development of obesity, with studies showing that early-life obesity risk factors, such as birthweight, maternal BMI, and rapid weight gain during infancy, are associated with variations in DNA methylation (DNAm) [19–21]. Given the role of epigenetics in obesity, nutritional epigenetics can play an important role in unravelling the effect of nutrition in the regulation of gene expression implicated in the aetiology of disorders such as obesity [22]. Recent epigenome-wide associations studies (EWAS) in adults found differentially methylated CpGs associated with diet quality, using measures of diet quality such as Mediterranean-style diet scores [23, 24]. A large proportion of these CpGs associated with diet quality have been linked to chronic diseases, in particular cardiometabolic diseases in adults [23, 24]. Other studies have investigated the intake of specific nutrients, such as fat and carbohydrate [25], or specific foods (coffee and tea) in relation to DNAm. However, the number of identified CpGs in these studies was comparatively smaller [26].

Existing diet-EWASs have been mainly focused on diet quality and were conducted in adults [23–26], newborns, or mothers [27, 28]. The few published EWASs in children so far have focused on glycaemic index [29] or on parental diet quality [30], but none have specifically examined UPF intake.

To explore the potential epigenetic changes associated with UPF consumption, we conducted the first meta-EWAS of UPF intake in relation to blood DNA methylation, in children aged between 5 and 11 years (middle childhood) from four European studies representing nine birth cohorts.

## Materials and methods

### Participating cohorts

We used data from four studies (Human Early-Life Exposome [HELIX], the Avon Longitudinal Study of Parents And Children [ALSPAC], Generation XXI, and Generation R) including a total of nine longitudinal birth cohorts that started with the recruitment of women during pregnancy or at delivery and followed up their children at different time points. The HELIX study comprises six sub-cohorts. All the children included were in overall healthy condition and did not have any congenital malformations.

The participants in cohorts from the HELIX sub-cohort were recruited between 2003 and 2010 from six birth cohorts across Europe [31]: BIB (Born in Bradford, UK [32, 33], EDEN (Study of determinants of pre- and postnatal development, France) [34], INMA (Environment and Childhood, Spain) [35], KANC (Kaunas Cohort, Lithuania) [36], MoBa (The Norwegian Mother and Child Cohort Study, Norway (Oslo region)) [37], and RHEA (Mother–Child Cohort in Crete, Greece) [38]. The follow-up period of HELIX children used in the present study was from 2013 to 2016 and included children between 5 and 11 years. All were of European ancestry, except in the BIB study which included 50% of children of South Asian ethnicity (n = 115). The Generation XXI (G21) study [39] recruited mothers living in the Porto metropolitan region, Portugal, in 2005–2006, and evaluated diet and blood DNAm of children of diverse ethnicity aged 9–11 years in 2015–2017. Mothers included in the ALSPAC study, in the UK [40, 41], were recruited between 1990 and 1992 with a total of 14,203 mothers, and we used the data of the children (G1 children) who attended examination at 7 years old. Finally, the Generation R (GenR) study was carried out in Rotterdam, included mothers recruited between April 2002 and January 2006. Child data used in the present study were from the follow-up when children were 10 years old [42].

Ethical approval for the study was granted by the Research Ethics Committees of each participating centre of each cohort. Full details are listed in the Supplementary Material S1.

### Dietary assessment

Dietary data of the HELIX children were collected via a semi-quantitative food frequency questionnaire (FFQ) covering the child’s habitual diet, which was filled in by the parent attending the examination appointment. The FFQ, developed by the HELIX research group, was translated to the corresponding country’s languages for each participating sub-cohorts of the HELIX project [31]. It included 43 questions about the consumption of various food and drink items [14].

Generation XXI dietary data were obtained from 2-day or 3-day food diaries (1 or 2 weekdays and 1 weekend day) filled by the main caregiver [43]. The codification process of food diaries was conducted by a team of trained nutritionists, using an age-specific food coding manual described elsewhere [44]. Energy and nutrient intakes were estimated using the software Food Processor SQL (2004–2005 ESHA Research) linked with the Food Composition Table of the US Department of Agriculture, and adapted with information of the Portuguese Food Composition Table at PortFir (http://portfir.insa.pt), and data from the European Food Information Resource (EuroFIR) network databases.

Dietary data in ALSPAC children were obtained using a 3-day food diary to record all food and beverage items the child consumed for 2 weekdays and 1 weekend day (not necessarily consecutive) which were completed by the caregivers [45]. Dietary data were reviewed by a nutritionist, and nutrient intakes were coded using the DIDO (Diet In, Data Out) computer program and linked to the 5th McCance and Widdowson British food composition tables [46]. Validity of dietary reporting was calculated using an individualized method based on the ratio of energy intake to estimated energy requirement and its 95% confidence interval.

In GenR, dietary intake was assessed using a validated 71-item semi-quantitative FFQ, completed by the parents of the child, reflecting the last 4 weeks of intake [47]. Information on frequencies, types, and portion sizes was converted into grams of individual food items per day based on standard Dutch portion sizes, using SAS VoVris (Vovris V2.4, TNO, 1999–2006).

To determine UPF intake, we identified foods and drinks as ‘ultra-processed’ by using the Nova classification (group 4), a food classification system based on the nature, extent, and purpose of industrial food processing [48]. To better capture dietary habits in each country, food group classifications are slightly different in each cohort, see Supplemental Table S1. The consumption of UPF for each child was defined as a continuous variable describing the intake of food group 4 relative to the total consumption of all food groups, either in weight (servings) or energy. In HELIX and GenR, UPF intake was expressed as the daily proportion of UPF serving (%) to the total daily sum of all food and drink servings as described in a recent article [14, 49], whereas in ALSPAC [15] and G21 [50], we used the proportion (%) of the total daily energy intake (kcal) derived from UPFs.

### DNA methylation

DNA methylation was measured in peripheral whole blood. HELIX, ALSPAC, and GenR used the Illumina Infinium HumanMethylation450 BeadChip array, which measures DNAm at 485,512 CpG sites and G21 used the Illumina Infinium MethylationEPIC BeadChip array that measures DNAm at 866,836 CpG. We used normalized beta values, ranging from 0 (fully unmethylated) to 1 (fully methylated). Further details on data normalization and quality control of DNAm in each cohort are given in Supplemental Material S2. In HELIX, ALSPAC, and G21, the array batch effect was corrected using the ComBat R package [51], while in GenR it was corrected by adjusting for sample plate. To reduce the influence of extreme methylation levels at individual samples in analysis, we winsorized methylation levels at each site. Winsorizing limits extreme values in a dataset and reduces the effect of spurious outliers by setting all data within the specified threshold. All the data below or above the 5th and 95th were set to those percentiles. White blood cell proportions (CD4 + and CD8 + T-cells, natural killer (NK) cells, monocytes, eosinophils, neutrophils, and B-cells) were estimated using the Houseman algorithm [52] and the Reinius reference panel [53].

### Covariates

The basic covariates considered were child’s age at time of follow-up, sex (categorized as male or female), and ethnicity (white European or non-white European children), assessed though questionnaires [10, 31–33, 49]. In the case of HELIX, most of the ethnicity heterogeneity comes from the BIB sub-cohort; therefore, HELIX models were adjusted by sub-cohort instead of ethnicity.

Maternal characteristics likely to affect either DNAm or diet related outcomes of the child [54, 55] were maternal age (in years) at birth (HELIX and G21) [31, 56] or at pregnancy (ALSPAC and GenR) [41, 57], maternal early-pregnancy body mass index (BMI) (kg/m^2^) with weight and height being measured, and smoking status during pregnancy (active smoking yes or no) as covariates. Maternal education in HELIX was defined as low, medium, and high [14]; in G21 based on the years of completed schooling as < 9y, 9-12y, and > 12y [49]; in ALSPAC as low (Certificate of Secondary Education, Vocational or Ordinary- (O-) level, educational qualifications generally obtained at 17 years of age), intermediate (Advanced- (A-) level (subject-specific qualifications generally obtained at age 18 years and required for university entry)), and high (university degree and above) [49]; in Gen R as low (no/primary education), intermediate (secondary school, vocational training), and high (bachelor’s degree, university) categories [42]. Maternal education was used as a proxy of socioeconomic status.

We also included as covariates: child’s BMI [58] and physical inactivity. BMI was calculated in kg/m^2^ based on weight (kg) and height (m^2^) measured at examination. Sedentary behaviour [59] was assessed by asking the carers about the minutes/day their child spent watching TV, playing on the computer, or other sedentary games in HELIX [14] and in GenR [60], as minutes/day without doing sports in G21 [49], and in ALSPAC categorized as 1 h, 1–2 h or > 3 h average time per day watching TV [49]. Vegetable and fruit intake (from the FFQ or food diaries) were also used as covariates as a proxy of overall diet quality. This was done to address if there are intrinsic associations of methylation with UPF independently of diet quality [61]. Vegetable intake was categorized as low (< 6 serving/week), medium (6–8.5/week), and high (> 8.5/week) and fruit intake as low (< 7 servings/week), medium (7–14.1 servings/week), and high (> 14.1 servings/week) in HELIX [31] and G21 [62]. In ALSPAC and in Gen R, vegetable and fruit intakes were continuous in grams/week [45, 47].

### Epigenome-wide association analysis (EWAS)

**Fig. 1:**
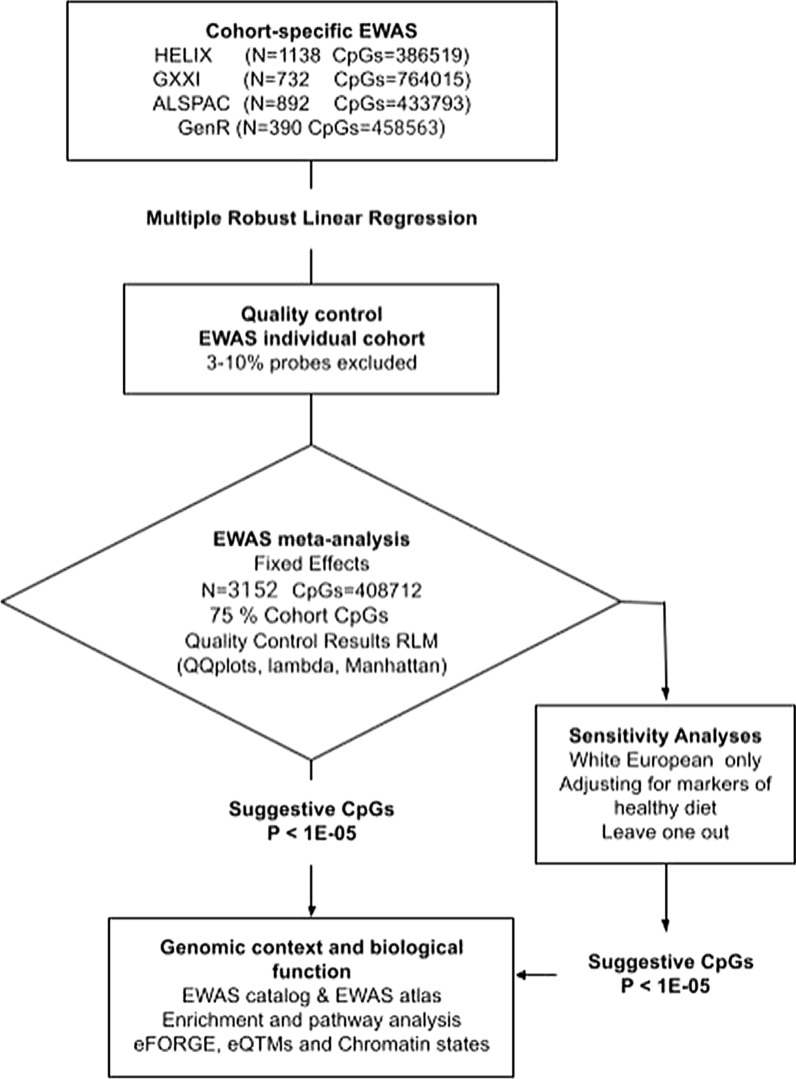
Flowchart of meta-EWAS

We included children who had available data on UPF intake (exposure) and whole-blood DNA methylation (outcome). UPF intake, covariates, and DNA methylation were assessed at the same time point, so this study has a cross-sectional design. Prior to conducting the analyses (Fig. 1), a data analysis plan including details of the variable definition, the statistical modelling, and a sample R code was distributed to the participating cohorts. All statistical analyses were carried out using R statistical software 4.3 version. To estimate the association between UPFs intake (exposure) and methylation at each CpG site (outcome), robust linear regression models (*rlm()* from MASS package) were employed. The models incorporated several covariates based on previous literature as described above and the Houseman 7-cell type proportions. In each cohort, nested models were fitted as follows:

1. Model 1: DNA methylation ~ UPF intake + HELIX sub-cohort or ethnicity + child sex + child age + cell type proportions
2. Model 2: model 1 + maternal age + maternal BMI + maternal smoking + maternal education
3. Model 3: model 2 + child BMI + child sedentary behaviour

In sensitivity analysis, we further adjusted for fruit and vegetable intake. Ethnicity was also considered, and children of non-European ancestry a minority in our study population were removed from the analysis (only in HELIX and G21) to ensure that any observed associations were not confounded by this factor. Therefore, two additional models were conducted:

• Model 3 + fruit intake + vegetable intake
• Model 3 restricted to white European ancestry children

### Quality control and meta-analysis

We performed the quality control of the cohort-specific EWAS results using the EASIER R package at ISGlobal (https://github.com/isglobal-brge/EASIER/). We excluded control probes, non-CpG probes, probes that mapped to X/Y chromosomes, probes with poor base pairing quality (lower than 40 on 0–60 scale), probes with non-unique 30 bp, subsequence (with cross-hybridizing problems), Infinium II probes with SNPs of global MAF over 1% affecting the extension base, and probes with a SNP in the extension base that causes a colour channel switch from the official annotation [63]. We also looked at the QQ plots as well as the inflation factor lambda (λ) and its confidence intervals.

To identify differentially methylated positions, EWAS results from each cohort were meta-analysed using inverse-variance weighted fixed-effects model. Heterogeneity was assessed using the I^2^ statistics [64] for each CpG site, considering low heterogeneity an I^2^ < 25%, medium 25– < 50%, and high ≥ 50%. We also performed random-effect model as sensitivity analyses. The meta-analysis was performed using GWAMA software in the *EASIER* package using the pcent parameter at 75% therefore only those probes that were available in at least 3 out of 4 cohorts were included in the meta-analysis. Only common probes for autosomal sites between the 450 K and EPIC arrays were considered, resulting in 408,712CpG sites to be tested in a total of 3152 children in the main analysis.

To account for multiple hypothesis testing, we applied the Benjamin–Hochberg false discovery rate (FDR) correction; an FDR-corrected *p* value < 0.05 denoted statistical significance [65]. A less stringent p-value threshold (pSuggestive) of *P* < 1 × 10^−5^ was used to select the top CpGs, as done in previous EWASs [66, 67]. Additionally, a shadow meta-analysis was conducted by an independent researcher using the *metafor* R package to confirm the obtained results. Finally, we conducted a leave-one-out analysis, in which we ran the main models with one of the cohorts removed each time, to explore if any of the studies was disproportionately influencing our results.

### Differentially methylated regions (DMRs)

To address the correlated structure of DNA methylation patterns, a regional analysis was conducted to identify differentially methylated regions (DMRs) associated with UPF intake in the main model in each individual cohort. The analysis was performed using the *dmrff* package in R, and all probes were included irrespective of their adjusted p-values. DMRs were defined based on the following criteria:

(i) Distance between two neighbouring probes was at most 500 base pairs.
(ii) The FDR-adjusted *p*-value for the region was < 0.05.
(iii) A DMR must include at least two CpG sites.

### Genomic and biologic context

We inspected the genomic surrounding of those CpGs that surpassed the suggestive threshold (*P* < 1 × 10^−5^) by looking at their annotation (i.e. chromosome, position, nearest gene, if any) according to the human genome assembly GRCh37/hg19 provided by Illumina R package. Additionally, to assess whether methylation levels of CpGs were associated with the expression levels of nearby genes in child blood, we consulted the HELIX Expression Quantitative Trait Methylation (eQTM) catalog and considered significant CpG-eQTM pairs those with a *p*-value < 10^–5^ [68].

To identify whether there were any existing associations between these CpG sites and exposures or health outcomes, we also consulted the EWAS Catalog [69]—a manually curated databases of CpG–trait associations (with *P*-value < 1 × 10^–4^) from published EWAS—and the EWAS atlas—a manually curated knowledgebase of EWAS associations (*P*-value < 1 × 10^–4^ or adjusted *P*-value < 0.05) [70].

### Functional enrichment analyses

To gain biological insight, we performed a series of functional enrichment analyses. To allow enough CpGs-genes to be included, a more lenient cut-off was used: *p*-value of 10^–4^ in model 3. Molecular functions and pathways that may be implicated in the associations between DNA methylation and high UPF intake were identified using Gene Ontology (GO) terms and pathways of the Kyoto Encyclopedia of Genes, Genomes (KEGG) using the missMethyl method [71], and protein complex-based sets (*P*) available in the Consensus Path database [72]. This was implemented in the Functional Enrichment module of the EASIER R package. We used eFORGE *version 2.0* [73] to test for enrichment of CpGs (*p*-value < 10^–4^) for DNase in hypersensitive sites, 15 chromatin states, and 5 histone marks in specific tissues and cells.

## Results

### Cohort characteristics

The characteristics of the participating cohorts are summarized in Table 1. Additionally, HELIX individual sub-cohort’s descriptive data are provided in Table S2. Our study population consisted of 1138 children from the HELIX study, 732 children from the G21 cohort, 892 children from the ALSPAC cohort, and 390 children from the GenR Study, resulting in a total of 3152 participants.

**Table 1:** Characteristics of mothers and children in all participating cohorts

N	Cohorts (N = 3152)			
	HELIX	G21	ALSPAC	GenR
	1138	732	892	390
Share of UPF in the diet in %^a^ (mean (SD))	24 (9)	34 (13)	61 (12)	36 (11)
Child sex: male (N (%))	627 (55.1)	392 (53.6)	442 (49.6)	200 (51.3)
Child age (mean (SD)) (years)	7.81 (1.55)	9.72 (0.23)	7.45 (1.68)	9.76 (0.26)
White European Ancestry (N (%))	1014 (89.1)	711 (97.1)	890 (99.8)	390 (100)
Child sedentary behaviour (min/day)^b^	240 (131)	492 (67)		124 (71)
Child sedentary behaviour (N (%))				
Screen time 1 h/day			246 (27.6)	
1-2 h/day			581 (65.1)	
3 h/day or more			65 (7.3)	
Child BMI (mean (SD)) (kg/m2)	16.8 (2.6)	18.6 (3.2)	16.2 (2.0)	17.1 (2.0)
Total vegetables intake (grams/week)^c^			197 (24)	98 (59)
Total vegetables intake (N (%))				
< 6 (servings/week)	573 (50.4)	188 (25.7)		
6.0–8.5 (servings/week)	216 (19.0)	346 (47.3)		
> 8.5 (servings/week)	349 (30.7)	119 (16.3)		
NA		79 (10.8)		
Total fruit intake (grams/week) ^d^			551 (513)	126 (68)
Total fruit intake(N (%))				
< 7 (servings/week)	372 (32.7)	387 (52.9)		
7–14.1 (servings/week)	384 (33.7)	207 (28.3)		
> 14.1 (servings/week)	382 (33.6)	59 (8.1)		
NA		79 (10.8)		
Maternal education (N (%)) ^e^				
Low	170 (14.9)	304 (41.5)	251 (28.1)	29 (7.4)
Middle	383 (33.7)	209 (28.6)	431 (48.3)	197 (50.5)
High	585 (51.4)	219 (29.9)	210 (23.5)	164 (42.1)
Maternal pre-pregnancy BMI (mean (SD)) (kg/m2)	25.0 (4.9)	24.5 (4.5)	22.9 (3.6)	24.6 (4.1)
Maternal age ^f^ (mean (SD)) (years)	30.7 (4.9)	29.6 (5.2)	29.7 (4.3)	42.4 (3.9)
Maternal active smoking during pregnancy (N (%))	168 (14.8)	153 (20.9)	161 (18.0)	47 (12.1)

^a^Share of UPF in the total diet was expressed as percentage of total energy derived from UPF in ALSPAC and G21, and percentage of servings to the total servings of food and drinks ingested in HELIX and GenR

^b^Sedentary behaviour in HELIX is defined as minutes/day spent watching a screen, in G21 as minutes/day without doing sport, GenR as minutes/day viewing television and computer game use

^c,d^Total vegetables and fruit intake in ALSPAC and in GenR is a continuous variable expressed in grams/week

^e^Maternal education in G21 is categorized in years of schooling (< 9 years, 9–12 years and > 12 years). The categories for maternal education in ALSPAC are: O level/vocational/CSE/no education qualifications, medium (A level), high (degree). The categories for GenR are: low (no/primary education), intermediate (secondary school, vocational training), and high (bachelor’s degree, university)

^f^Maternal age at birth for HELIX, G21, and ALSPAC and at child assessment for GenR

All continuous variables were expressed as mean ± SD while categorical variables are expressed as numbers, n (%)

The average age was between 5 and 11 years overall. Most of the children were of white European ancestry (95%). The distribution of UPFs intake varied across the study cohorts. UPF intake contributed on average 24% in HELIX and 36% in GenR to the total food consumption in servings, and 34% in G21 and 61% in ALSPAC to total energy intake. Mothers were on average 30 years old at the time of pregnancy in all cohorts and their early-pregnancy BMI ranged from ~ 22 kg/m^2^ in ALSPAC to ~ 25 kg/m^2^ in HELIX, G21, and GenR. We observed the lowest percentage of highly educated mothers in ALSPAC (23%) and the highest in HELIX (51%). Maternal smoking during pregnancy ranged from 12% in GenR to 20% in G21. All cohorts have complete case data except for G21, where there is a 10.8% of missing data on fruit and vegetable intake.

### Epigenome-wide association study meta-analysis

The percentage of probes excluded in each cohort ranged between 3 and 10% (Table S3). The lambdas (measure of p-value inflation) of different models in each cohort and in the meta-analysis ranged from 0.97 to 1.01 (Table S4).

No CpG reached the FDR significance threshold in association with UPF intake (see Manhattan plot Figure S1). However, as reported in Table 2, four CpGs reached the suggestive significance level (*p*-value < 10^–5^) with low heterogeneity between cohorts (I^2^ < 50%) and consistent direction of effect. These results are also consistent with the random-effect model, as shown in Table S5.

**Table 2:** Fixed effect meta-analysis for the top CpGs associated with UPF intake in children

Model	Beta	Standard error	p-value	Estimate direction (HELIX, G21, ALSPAC, GenR)	I2 (%)
cg14665028					
Model 1	0.0011	0.0003	4.3E−05	+ + + +	11.1
Model 2	0.0011	0.0003	1.8E−05	+ + + +	0.0
Model 3	0.0012	0.0003	5.3E−06	+ + + +	0.0
Sensitivity 1	0.0012	0.0003	2.7E−05	+ + + +	0.0
Sensitivity 2	0.0011	0.0003	1.3E−04	+ + + +	0.0
cg18968409					
Model 1	0.0057	0.0011	4.5E−07	+ + + +	0.0
Model 2	0.0056	0.0011	9.3E−07	+ + + +	0.0
Model 3	0.0057	0.0012	9.4E−07	+ + + +	0.0
Sensitivity 1	0.0061	0.0012	3.6E−07	+ + + +	0.0
Sensitivity 2	0.0059	0.0012	1.3E−06	+ + + +	0.0
cg00339913					
Model 1	− 0.0034	0.0008	9.7E−06	− − − −	0.0
Model 2	− 0.0036	0.0008	4.1E−06	− − − −	0.0
Model 3	− 0.0036	0.0008	7.0E−06	− − − −	0.0
Sensitivity 1	− 0.0034	0.0008	3.0E−05	− − − −	0.0
Sensitivity 2	− 0.0039	0.0008	2.2E−06	− − − −	0.0
cg24730307					
Model 1	0.0011	0.0003	2.3E−05	+ + + +	0.0
Model 2	0.0011	0.0003	2.0E−05	+ + + +	0.0
Model 3	0.0012	0.0003	4.8E−06	+ + + +	0.0
Sensitivity 1	0.0012	0.0003	6.2E−06	+ + + +	0.0
Sensitivity 2	0.0012	0.0003	2.3E−05	+ + + +	0.0
cg03041696					
Model 1	− 0.0017	0.0003	1.4E−06	− − − −	4.9
Model 2	− 0.0016	0.0003	3.3E−06	− − − −	2.4
Model 3	− 0.0016	0.0004	2.2E−05	− − − −	8.7
Sensitivity 1	− 0.0016	0.0004	7.8E−06	− − − −	6.8
Sensitivity 2	− 0.0017	0.0004	4.7E−06	− − − −	0.0
cg09709951					
Model 1	0.0053	0.0013	3.5E−05	+ + + ?	0.0
Model 2	0.0055	0.0013	2.8E−05	+ + + ?	0.0
Model 3	0.0059	0.0013	1.2E−05	+ + + ?	0.0
Sensitivity 1	0.0062	0.0014	5.9E−06	+ + + ?	0.0
Sensitivity 2	0.0063	0.0014	6.6E−06	+ + + ?	0.0
cg03999434					
Model 1	− 0.0015	0,0004	6.8E−05	− − − −	45.6
Model 2	− 0.0017	0.0004	2.0E−05	− − − −	37.2
Model 3	− 0.0017	0.0004	2.2E−05	− − − −	36.6
Sensitivity 1	− 0.0018	0.0004	7.1E−06	− − − −	28.9
Sensitivity 2	− 0.0016	0.0004	7.1E−05	− − − −	34.0

Model 1 includes basic potential confounders related to the child (ethnicity, age, and sex)

Model 2 extends adjustments to maternal variables like smoking, education level, maternal BMI and age

Model 3 (main model) further includes child's sedentary behaviour and BMI

Sensitivity 1: Model 3 restricted to European children only (n = 3007) (excluding non-White children); Sensitivity 2: Model 3 + further adjusting for fruit and vegetable consumption

Beta values are DNA methylation change per 1% increase in UPF consumption

Two of these suggestive CpGs were annotated to genes: (cg14665028, annotated to *NHEJ1* on chromosome 2 and cg00339913 annotated to *PHYHIP* on chromosome 8), while the other two were in an intergenic region: cg18968409 on chromosome 2 and cg24730307 on chromosome 22. Forest plots for the associations of these 4 CpGs with UPF intake across the 4 cohorts are presented in Fig. S2.

No statistically significant (FDR-adjusted *P* > 0.05) or suggestive (*p*-value < 10^–5^) DMRs were found.

### Sensitivity analysis

First, when restricting the meta-analysis to children of European ancestry (n = 3007), most estimates remained similar, although the magnitude was slightly stronger for cg18968409 (effect size). Three additional CpGs passed the indicative threshold of *p* < 10^–5^: cg09709951, annotated to *ATF7,* cg03999434 (intergenic) and cg03041696 (intergenic) (Table 2). Secondly, when further adjusting for fruit and vegetable intake, we found suggestively significant associations for 4 CpGs, two of them previously detected in model 3 ((cg18968409 and cg09709951 (*ATF7*)) and two CpGs identified in the ethnicity sensitivity analyses (cg03999434 and cg03041696). Therefore, additionally to the 4 CpGs identified in model 3, we detected 3 additional CpGs in the two sensitivity analyses. Forest plots of these 3 additional CpGs are also included in Figure S2.

In leave-one-out analyses, estimates remained fairly consistent and there was no strong evidence that a specific cohort disproportionately influenced findings (Table S6).

### Genomic and biological context

Table 3 shows genomic characteristics of the 7 suggestion CpGs and previously identified associations of DNAm with other exposures or traits in various age populations. Focusing on the genomic context, out of 7 CpGs, only 3 were annotated to a gene, of which only one (cg09709951) was associated with two transcript clusters: TC12002893.hg.1 and TC12001553.hg.1, which encode for the nearby *ATF7* gene. As for the biological context, according to the EWAS catalog, 5 of the 7 identified CpGs had previously been reported in relation to ageing from birth to adolescence [74]. Moreover, natural juice consumption has been inversely associated with methylation at cg09709951 in adult studies [75], while UPF is associated with higher methylation at this site in our study, including when adjusting for fruit and vegetable intake. Lower methylation levels in the liver have been observed at cg03999434 (transcript: long non-coding RNA, RP3-340I3.1) in foetus compared to adults [76]. In the EWAS atlas, lower methylation at cg00339913 and higher methylation at cg18968409 have been associated with different carcinomas in adults [77, 78]. Also, higher methylation at cg09709951 has been found to be related to neurodevelopmental disorder related to SETD1B gene in children [73]. The directions of these associations with methylation correspond to a higher UPF intake in our study.

**Table 3:** Genomic context and biological exposures and traits previously associated with suggestive CpGs

CpG ID (UPF association)	Chromosome	Position	Relation to island	Gene region	Nearest gene	eQTM ^a^	EWAS catalog (tissue exposure direction) ^b^	Ewas atlas (biological traits) ^c^
cg14665028 ( +)	2	220,024,924	Island	5'UTR	*NHEJ1*	0	Buccal cells tissue versus blood [95]	No results
cg18968409 ( +)	2	34,633,637	Open Sea			0	Whole blood: age (−) [74]	Adults: hepatocellular carcinoma HCC ( +) [78]
cg00339913 (−)	8	22,085,227	Open Sea	Body	*PHYHIP*	0	Whole blood: age (−) [74]	Papillary thyroid carcinoma (−) [77]
cg03041696 (−)	14	21,094,001	S Shore			0	Whole blood: age (−) [74]	Mother–newborns: alcohol consumption ( +) [96]
cg24730307 ( +)	22	24,405,009	N Shelf			0	Buccal cells tissue vs blood [95]	No results
cg09709951 ( +)	12	54,017,699	N Shelf	5'UTR	*ATF7*	2 *(ATF)*	Whole blood: age ( +)[74] sex ( +)[74] Adults: juice consumption (−) [75]	SETD1B-neurodevelopment-related syndrome ( +) [73]
cg03999434 (−)	12	1,639,034	Island			0	Whole blood: age ( +)[74] sex (−) [74] Liver: adult liver versus foetus (−) [76]	Adults: asthma (−) [97] air pollution (−) [98] Children: age (−) [99]

^a^Expression Quantitative Trait Methylation (eQTMs) associated with each CpGs, derived from the HELIX eQTM blood database

^b^Reported associations in the EWAS catalog [37]. All are in children, except when indicated otherwise

^c^Reported associations with biological traits documented in the EWAS atlas [63]

When searching EWAS of diet at any life stages, we did not find our suggestive CpGs (*p*-value < 1 × 10^–5^) to be reported in existing studies (Table S7). However, 16 of the CpGs reported in these studies were associated with UPF at a nominal *p*-value (*p* < 0.05) in our analyses (Table S8).

### Functional enrichment analyses

We found some enriched pathways in the protein complex set of Consensus Path (Table 4). Three of the identified pathways are related to thyroid hormone, two others related to cancer and one related to LKB1 gene, which encodes a serine/threonine kinase protein that regulates cell polarity and energy metabolism and functions as a tumour suppressor [79].

**Table 4:** Protein complex gene sets annotated to CpGs associated with UPF with a *p* value < 10^–4^

TERM^a^	N^b^	DE^c^	*P*	FDR
Regulation of apoptosis by parathyroid hormone-related protein	2	21	< 0.001	0.002
*LKB1* signalling events (PID)	2	43	0.001	0.005
Thyroid hormones production and their peripheral downstream signalling effects	2	74	0.002	0.010
Fragile X syndrome	2	119	0.002	0.010
Thyroid hormone signalling pathway	2	121	0.004	0.015
Transcriptional deregulation in cancer	2	192	0.010	0.031
MicroRNAs in cancer--Homo sapiens (human) (KEGG)	2	310	0.025	0.061
PI3K-Akt signalling pathway	2	340	0.030	0.061
PI3K-Akt signalling pathway	2	354	0.032	0.061
Transcriptional Regulation by TP53	2	361	0.034	0.061

^a^Term from complex gene sets from ConsensusPathDB[66]

^b^Number of genes of UPF-related CpGs overlapping with the genes annotated to the ConsensusPathDB term

^c^Total number of genes in the ConsensusPathDB term

We did not find any enriched GO or KEGG terms that surpassed the FDR significance threshold; however, a list of the top enriched terms with a nominal *p* value < 0.05 is shown in Table S9. The first three nominal enriched GO terms are related to transcription processes, followed by transaminase-related terms, which are involved in the GABA catabolism, an inhibitory neurotransmitter [80]. In KEGG, we identified pathways related to thyroid hormone signalling, DNA repair, and cancer, the latter aligning with previous associations found with carcinomas. Some nominal KEGG enriched terms are also related to amino acid metabolism and energy metabolism which is related to gut microbiota and also is involved in GABA metabolism.

No specific enriched tissue or cell type for any chromatin states was found significant (Table S11).

## Discussion

In this exploratory study, we report the first meta-EWAS of UPF intake and DNA methylation in blood in children in middle childhood (5–11 years). No CpG reached the FDR significance threshold in association with UPF intake; however, we found 7 suggestive CpGs (*p* < 10^–5^) associated with UPF consumption. The functional analysis reveals some enriched pathways involved in thyroid and liver function.

### Biological interpretation and association with health outcomes

We observed higher methylation levels at cg09709951 (*ATF7*) associated with UPF intake, whereas the literature reports lower methylation with juice consumption in adults [75]. Our results align that quality of the diet might modulate methylation at this CpG, with a lower diet quality associated with higher methylation, although fruit juice is not similar to fruit in terms of fibre content and glycaemic load, and on its health effects. Moreover, higher methylation of this CpG has been associated with a neurodevelopmental disorder in children [81]. Finally, ATF7 encodes a stress-responsive chromatin regulator that plays a role in various biological processes including innate immunological memory, adipocyte differentiation, and telomerase regulation [82]. Some studies have shown that ATF7 ablation prevents diet-induced obesity and insulin resistance [82].

Maternal characteristics have also been associated with some of the suggestive CpGs; for instance, maternal alcohol consumption has been associated with a higher methylation at cg03041696 [74] (opposite direction with UPF), while lower methylation of cg00339913 (*PHYHIP*) has been associated with higher maternal BMI and overweight [20], following the same direction as UPF consumption. Our analyses were adjusted for maternal and child BMI; therefore, the differential methylation at this CpG previously observed with maternal BMI might be at least partly explained by consumption of UPF in the diet.

Some of our results point towards implications of UPF-related CpGs in liver function pathways. First, higher methylation at cg18968409 linked to higher UPF intake was also observed in the presence of hepatocellular carcinoma [78]. Second, cg03999434 is reported to be less methylated in the liver in the foetus compared to adults [76] and methylation is also expected to increase in blood at this CpG from birth to adulthood, whereas UPF is associated with lower methylation levels in our study. Additionally, pathway analyses revealed a protein complex near the *LKB1 gene*, which in the liver tissue is involved in the glycogenesis pathway. In adults, UPF intake has been related to adverse liver outcomes, in particular non-alcoholic fatty liver disease [83], cirrhosis, and severe liver disease [84]. Studies in children are needed to investigate if this starts in early life, particularly with subclinical biomarkers such as liver enzymes.

We found that UPF intake was associated with higher methylation of cg14665028, located at the promoter of *NHEJ1* gene. This gene is one of the main genes involved in the non-homologous end-joining (NHEJ) pathway, the major double-strand breaks (DSB) repair mechanisms in normal cells [85]. This gene has been associated with a negative correlation with DNAm in tumour tissues [85], and an increase in DNA damage has been described in mice fed with high-fat diet and knockout in NHEJ pathway [86].

UPF consumption was inversely associated with methylation at cg00339913, located at the gene body of *PHYHIP*, also named *PAHX-AP1,* a brain specific protein mainly related to early postnatal maturation of visual function [87]*.* Lower methylation at this CpGs has been associated with presence of papillary thyroid carcinoma in non-neoplastic adjacent tissue in comparison to PTC tissue [77].

### UPF intake and health outcomes

Despite conducting our EWAS on a very large multi-cohort population, the identified associations between UPF intake and DNA methylation in our study are of weak magnitude. UPF intake is associated with a range of health outcomes [8], some of them related to the UPF-related CpGs and pathways identified here, in particular thyroid and liver function. For instance, studies have reported that some food additives are related to thyroid hormone dysregulation [88] and an increased risk of subclinical thyroid dysfunction [88]. A recent report on the HELIX data showed that metabolites associated with UPF intake were associated with C-peptide levels in children, a well-known marker of β-cell function and insulin resistance. However, a wide range of non-methylation mechanisms that include the gut microbiome, oxidative stress, and inflammation are likely to be more affected by UPF intake and by the contaminants (acrylamide, etc.) and additives (emulsifiers, sweeteners, etc.) that they contain. Although UPFs are typically high in at least one nutrient of concern (saturated fat, salt, added sugar), Nova classification classifies UPFs based on the extent of food processing and not on nutrient profiles [89].

### Limitations and strengths

This study provides initial evidence for the possible association of UPF consumption with DNA methylation in children. However, these results should be interpreted considering both the limitations and strengths of the study.

A first limitation is the cross-sectional design, which limits causal inference of the observed associations.

However, we carefully selected potential confounders and provide estimates at various levels of adjustment to reduce the likelihood of confounding. Additionally, our results are confined to the 'middle childhood' period (5–11 years) [90] and are not generalizable to younger ages or to adolescents. Moreover, it is important to acknowledge that both dietary and methylation patterns are likely to evolve from the age of 5 to 11 and that the association between UPF consumption and DNA methylation may vary across different ages.

Secondly, the definition of the exposure as percentage of UPF of the total dietary intake was defined differently across cohorts: proportion in servings in HELIX and GenR, and proportion of energy intake in ALSPAC and Gen XXI. Despite these definition differences between studies (a limitation inherent to any meta-analysis), the top hits CpGs were all associated with UPF in the same direction and with a similar magnitude across cohorts. Moreover, it should be noted that UPF intake calculated using the same definition in HELIX was associated with metabolites associated with insulin resistance and inversely related with a Mediterranean diet index [14].

In addition, our study considers geographical specificities and some foods were classified as UPF in some cohorts, but not in others. In particular, bread is most commonly purchased in supermarkets (cooked from ready-made industrial frozen dough, sliced packaged bread) in the UK and was therefore classified as ultra-processed in the ALSPAC study, whereas in the other countries involved in this study (Portugal, Spain, France, Lithuania, Greece and the Netherlands), fresh bread is most commonly consumed and therefore not classified as UPF [91].

Our results report methylation levels in whole blood. However, since DNA methylation is tissue specific, our findings in whole blood may not reflect DNA methylation levels in other tissues, where such associations may be more evident. For instance, gastrointestinal tissue, responsible for food digestion nutrient extraction, absorption, and waste excretion, could be a more appropriate tissue to provide a more relevant context [92]. Considering that UPF have been linked to metabolic biomarkers [14] and that the microbiome contains genomic information shaped by factors such as age and diet [20], it would be of interest to detect associations between diet and UPF within the gastrointestinal tissue DNA methylation. This may also be true for the liver tissue—as methylation may be related to some pathways such as gluconeogenesis [93]—the thyroid or the adipose tissue [94].

Despite the effort to combine various well characterized European children cohorts to increase power, the magnitude of the associations between DNA methylation and UPF consumption observed was small. A possible explanation is that the life-long cumulative exposure to UPF is shorter compared to adults, which might explain the lower number of CpGs related to diet in previous EWASs conducted in children too [29, 30]. This absence of clear results also likely comes partly from the inaccuracy of dietary assessment preventing the discovery of larger effects. Well-designed intervention studies that aim at reducing UPF intake and increase diet quality are needed to identify more robust effects of diet on DNA methylation, in particular if this is measured in relevant tissues beyond blood.

## Conclusions

This study constitutes the first meta-EWAS evaluating the association between child UPF consumption and differential DNA methylation in blood in over 3000 school-aged children across Europe. We found suggestive changes in methylation at 7 CpGs. Future research with larger sample sizes, more detailed dietary intake estimation, and additional epigenetic time points will help to clarify the epigenetic signature of UPF intake.

## Supplementary Information

Additional file1

## Data Availability

The manuscript is based on the data from the 4 European birth cohort studies (HELIX, Generation XXI, ALSPAC and GenerationR). The data that support the finding of this study are available from the four cohort, but restrictions apply to the availability of these data, which were used under licence for the current study and so are not publicly available. The HELIX cohort available data can be found in the codebooks publicly available at the project website (https://www.projecthelix.eu/index.php/es/data-inventory). The ALSPAC data are available through a fully searchable data dictionary and variable search tool (http://www.bristol.ac.uk/alspac/researchers/our-data/). All data are available on request for all participating cohort. The R scripts are available here: https://github.com/joanall/EWAS_UPF.
