# Supplementary material for: A meta-analysis of epigenome-wide association studies of ultra-processed food consumption with DNA methylation in European children
Source: Clin Epigenetics. 2025 Jan 7;17:3. doi: 10.1186/s13148-024-01782-z (PMC11706074; doi:10.1186/s13148-024-01782-z)
Supplement: Supplementary file 1 — Additional file1 [file 13148_2024_1782_MOESM1_ESM.pdf]

Supplementary information to

A meta-analysis of epigenome-wide association studies of ultra-processed food consumption with DNA methylation in European children

Llauradó-Pont J, et al. *Clinical Epigenetics* doi: 10.1186/s13148-024-01782-z

Table of content

Supplementary material S1. Consent and ethical approval
Supplementary material S2. DNAm Quality Control and normalization
Supplementary material S3. Funding

Table S1. Items included in the UPF classification in each cohort
Table S2. Descriptive characteristics in the individual cohorts of the HELIX project
Table S3. Quality Control EWAS individual cohorts
Table S4. Inflation factor lambda and 95% CI for each adjustment model, Fixed Effects Meta-analysis
Table S5. Random effect meta-analysis for the top CpGs associated with UPF intake in children.
Table S6. Leave-one-out analysis: fixed effect meta-analysis of the 7 suggestive CpGs ($p<10^{-5}$), identified in Model 3 in meta-analysis across 4 cohorts
Table S7. List of studies and CpGs reported to be associated with diet
Table S8. List of diet-related CpGs replicated in our study with a nominal p value (p < 0.05)
Table S9. Suggestive CpGs (p-value<10-4) associated with UPF, and list of CpGs and genes used in enrichment analyses (Model 3)
Table S10. Top 10 of enriched pathways from GO and KEGG gene sets with a nominal p-value < .05
Table S11. Top 10 Enriched tissue with a nominal p value < 0.05

Figure S1: Manhattan plot of the fixed effect meta-EWAS of UPF intake (Model 3)
Figure S2. Forest plots of the 7 suggestive CpGs associated with UPF intake across the 4 studies included in the meta-analysis (Model 3)

Supplementary material S1. Consent and ethical approval

Human Early Life Exposome (HELIX)

All six cohorts on which HELIX is based have undergone the required evaluation by national ethics committees and have obtained all the required permissions for their cohort recruitment and follow-up visits. Each cohort also confirmed that relevant informed consent and approval were in place for secondary use of data from pre-existing data. The work in HELIX was covered by new ethics approvals in each country, and at enrolment in the HELIX sub-cohort and panel studies participants were asked to sign an informed consent form for the specific HELIX work. All data exchanges will adhere to the most up-to-date EU and national data protection regulations.

Generation XXI (G21)

Generation XXI was approved by the Portuguese Data Protection Authority and by the Ethics Committee of Hospital São João, and data confidentiality and protection were guaranteed in all procedures according to the Declaration of Helsinki. Informed consent was obtained for all participants, signed by their legal guardian at every study wave.

Avon Longitudinal Study of Parents and Children (ALSPAC)

Informed consent for the use of data collected via questionnaires and clinics was obtained from participants following the recommendations of the ALSPAC Ethics and Law Committee at the time. Children were invited to give assent where appropriate. Study participants have the right to withdraw their consent for elements of the study or from the study entirely at any time. Full details of the ALSPAC consent procedures are available on the study website.

Generation R

The general design, all research aims and the specific measurements in the Generation R Study have been approved by Erasmus MC Center in close collaboration with the School of Law and Faculty of Social Sciences of the Erasmus University Rotterdam. At the start of each phase, children and their parents receive written and oral information about the study and consent is obtained for all participants.

Supplementary material S2. DNAm Quality Control and normalization

Human Early Life Exposome (HELIX)

DNA methylation data was pre-processed using the minfi package [1] and probes not reaching a 98% call rate were excluded [2]. Data was normalized with the functional normalization method, which also includes Noob background subtraction and dye-bias correction [3]. Sex consistency was also checked using the shinyMethyl package [4]. Genetic consistency of duplicates and samples from the same participant was checked with the 450k genotypes. Finally, duplicated samples and HapMap samples were removed as well as control probes, probes designed to detect SNPs and probes to measures methylation levels at non-CpG sites. The final dataset consisted of 386,518 probes.

Generation XXI (G21)

Generation 21 is funded by Saude XXI (Programa Operacional Saude), Administração Regional de Saúde do Norte (ARS NORTE), Fundação para a Ciência e a Tecnologia, and Fundação Calouste Gulbenkian. DNA methylation experiments for G21 are funded by the 'Lifepath' H2020 research grant to Paolo Vineis and Henrique Barros (n. 633666).

Avon Longitudinal Study of Parents and Children (ALSPAC)

Avon Longitudinal Study of Methods for methylation measurements in ALSPAC have been described previously [5] . Briefly, samples failing QC (average probe P-value ≥0.01) were excluded from further analysis. As an additional QC step, genotype probes were compared with SNP-chip data from the same individual to identify and remove any sample mismatches. For individuals with no genome-wide SNP data, samples were flagged if there was a sex-mismatch based on X-chromosome methylation. Background correction and subset quantile normalization was performed within each time point using the pipeline described by Touleimat and Tost [5]

Generation R

Preparation and normalization of the Illumina Infinium® HumanMethylation450 BeadChip array data was performed according to the CPACOR workflow using the software package R. Probes that had a detection p-value above background (based on sum of methylated and unmethylated intensity values) > or equal to 1E-16 were set to missing per array. Next, the intensity values were stratified by autosomal and non-autosomal probes and quantile normalized for each of the six probe type categories separately:

type II red/green, type I methylated red/green and type I unmethylated red/green. Arrays with observed technical problems such as failed bisulfite conversion, hybridization or extension, as well as arrays with a mismatch between sex of the probe and sex determined by the chromosome X and Y probe intensities were removed from subsequent analyses. Additionally, only arrays with a call rate > 95% per sample were processed further. The final dataset contained information on 458,563 CpGs

References

[1] S. A. Islam *et al.*, "Integration of DNA methylation patterns and genetic variation in human pediatric tissues help inform EWAS design and interpretation," *Epigenetics Chromatin*, vol. 12, no. 1, p. 1, Dec. 2019, doi: 10.1186/s13072-018-0245-6.

[2] B. Lehne *et al.*, "A coherent approach for analysis of the Illumina HumanMethylation450 BeadChip improves data quality and performance in epigenome-wide association studies," *Genome Biol.*, vol. 16, no. 1, p. 37, Dec. 2015, doi: 10.1186/s13059-015-0600-x.

[3] T. J. Triche, D. J. Weisenberger, D. Van Den Berg, P. W. Laird, and K. D. Siegmund, "Low-level processing of Illumina Infinium DNA Methylation BeadArrays," *Nucleic Acids Res.*, vol. 41, no. 7, pp. e90–e90, Apr. 2013, doi: 10.1093/nar/gkt090.

[4] J.-P. Fortin, E. Fertig, and K. Hansen, "shinyMethyl: interactive quality control of Illumina 450k DNA methylation arrays in R," *F1000Research*, vol. 3, p. 175, Sep. 2014, doi: 10.12688/f1000research.4680.2.

[5] C. L. Relton *et al.*, "Data Resource Profile: Accessible Resource for Integrated Epigenomic Studies (ARIES)," *Int. J. Epidemiol.*, vol. 44, no. 4, pp. 1181–1190, Aug. 2015, doi: 10.1093/ije/dyv072.

[6] N. Stratakis *et al.*, "Urinary metabolic biomarkers of diet quality in European children are associated with metabolic health," *Elife*, vol. 11, p. 71332, Jan. 2022, doi: 10.7554/ELIFE.71332.

[7] G. M. Vedovato, S. Vilela, M. Severo, S. Rodrigues, C. Lopes, and A. Oliveira, "Ultra-processed food consumption, appetitive traits and BMI in children: A prospective study," *Br. J. Nutr.*, vol. 125, no. 12, pp. 1427–1436, Jun. 2021, doi: 10.1017/S0007114520003712.

[8] K. Chang *et al.*, "Association between Childhood Consumption of Ultraprocessed Food and Adiposity Trajectories in the Avon Longitudinal Study of Parents and Children Birth Cohort," *JAMA Pediatr.*, vol. 175, no. 9, pp. 1–11, Sep. 2021, doi: 10.1001/jamapediatrics.2021.1573.

[9] L. A. Van Der Velde *et al.*, "Diet quality in childhood: the Generation R Study," *Eur. J. Nutr.*, vol. 58, pp. 1259–1269, 2019, doi: 10.1007/s00394-018-1651-z.

Supplementary material S3. Funding

The HELIX cohort received funding from the European Community's Seventh Framework Programme (FP7/2007–2013) under grant agreement 308333. **INMA** data collections were supported by grants from the Instituto de Salud Carlos III, CIBERESP, the Conselleria de Sanitat, Generalitat Valenciana, Department of Health of the Basque Government; the Provincial Government of Gipuzkoa, and the Generalitat de Catalunya-CIRIT. **KANC** was funded by the grant of the Lithuanian Agency for Science Innovation and Technology (6-04-2014_31V-66). The **Norwegian Mother, Father and Child Cohort Study** is supported by the Norwegian Ministry of Health and Care Services and the Ministry of Education and Research. The **Rhea** project was financially supported by European projects, and the Greek Ministry of Health (Program of Prevention of Obesity and Neurodevelopmental Disorders in Preschool Children, in Heraklion district, Crete, Greece: 2011–2014; 'Rhea Plus': Primary Prevention Program of Environmental Risk Factors for Reproductive Health, and Child Health: 2012–2015). The work was also supported by MICINN (MTM2015-68140-R) and Centro Nacional de Genotipado-CEGEN-PRB2-ISCIII. The **EDEN** study was supported by Foundation for Medical Research (FRM), National Agency for Research (ANR), National Institute for Research in Public health (IRESP: TGIR cohorte santé 2008 programme), French Ministry of Health (DGS), French Ministry of Research, INSERM Bone and Joint Diseases National Research (PRO-A) and Human Nutrition National Research Programs, Paris-Sud University, Nestlé, French National Institute for Population Health Surveillance (InVS), French National Institute for Health Education (INPES), the European Union FP7 programmes (FP7/2007–2013, HELIX, ESCAPE, ENRIECO, Medall projects), Diabetes National Research Program (through a collaboration with the French Association of Diabetic Patients (AFD)), French Agency for Environmental Health Safety (now ANSES), Mutuelle Générale de l'Education Nationale complementary health insurance (MGEN), French national agency for food security, French-speaking association for the study of diabetes and metabolism (ALFEDIAM). **BiB** received funding from a Wellcome Infrastructure Grant (WT101597MA) and a joint grant from the UK Medical Research Council (MRC) and UK Economic and Social Science Research Council (ESRC) (MR/N024391/1)

The UK Medical Research Council and Wellcome (Grant ref: 217065/Z/19/Z) and the University of Bristol provide core support for ALSPAC. Methylation data in the ALSPAC cohort were generated as part of the UK BBSRC funded (grant numbers: BB/I025751/1 and BB/I025263/1) Accessible Resource

for Integrated Epigenomic Studies (ARIES, http://www.ariesepigenomics.org.uk). Unit that is supported by the University of Bristol and the UK Medical Research Council (grant number: MC_UU_00011/5).

G21 was funded by the Programa Operacional de Saúde – Saúde XXI, Quadro Comunitário de Apoio III (Health Operational Programme – Saúde XXI, Community Support Framework III) and the Administração Regional de Saúde Norte (Regional Department of Ministry of Health). It was also supported by FCT—Fundação para a Ciência e Tecnologia, I.P. (Portuguese Ministry of Science, Technology and Higher Education) through the projects with references UIDB/04750/2020 and LA/P/0064/2020 and DOI identifiers https://doi.org/10.54499/UIDB/04750/2020 and https://doi.org/10.54499/LA/P/0064/2020

Generation R Study (Generation R): The general design of the Generation R Study is made possible by financial support from Erasmus MC, Erasmus University Rotterdam, the Netherlands Organization for Health Research and Development and the Ministry of Health, Welfare and Sport. The EWAS data were funded by a grant from the Netherlands Genomics Initiative (NGI)/Netherlands Organisation for Scientific Research (NWO) Netherlands Consortium for Healthy Aging (NCHA; project nr. 050–060-810), by funds from the Genetic Laboratory of the Department of Internal Medicine, Erasmus MC, and by a grant from the National Institute of Child and Human Development (R01HD068437).

This project received funding from the European Union’s Horizon 2020 research and innovation programme (874739, LongITools; 874583, ATHLETE; 824989, EUCAN-Connect; 774548, STOP Project) and from the European Joint Programming Initiative 'A Healthy Diet for a Healthy Life' (JPI HDHL, NutriPROGRAM project, ZonMw the Netherlands no.529051022 and PREcisE project ZonMw the Netherlands no.529051023).

ISGlobal acknowledges support from the Spanish Ministry of Science and Innovation and State Research Agency through the “Centro de Excelencia Severo Ochoa 2019–2023” Program [CEX2018-000806-S] and Fondo Europeo de Desarrollo Regional, UE [PID2021-122855OB-I00], and support from the Generalitat de Catalunya through the CERCA Program and Ministery of Research and Universities [2021 SGR 01563].

Table S1. Items included in the UPF classification in each cohort

**HELIX** [6]	**Generation XXI** [7]	**ALSPAC** [8]	**GenR** [9]
**Foods in the UPF group**			
Cookies and pastries, Chocolate and Sweets	Cookies, pastries and cake mixes, Chocolate and Candies	Cakes and desserts, Confectionery, Jams, spreads and sweet sauces, Packaged sweet snacks	Sweets, sweet snacks and cookies
Sugar-Sweetened Beverages, artificially-sweetened and low-sugar beverages	Soft drinks	Fruit juice beverages, Carbonated beverages, Others (Flavoured water, artificial sweetener)	Lemonades, sugar and low-sugar, lemonade concentrate and fruit juice concentrates, soda, soft drinks
Cold meat cuts, Ham	Processed meat and sausages	Sausages and reconstituted meat products	Processed Red and white Meat
Dairy dessert/ milky pudding, ice cream	Ice Cream, Milk, cocoa and fruit drinks, Flavoured and/or artificial sweetened yoghurt	Yogurt and dairy-based drinks	Dairy based desserts (including ice cream), soy milk / soy dessert, milk-based beverages, yoghurt or quark with added sugar
Sugar sweetened breakfast cereals Other breakfast cereals (porridge)	Breakfast cereals and cereal/energy bars	Breakfast cereals	Cereals with white sugar
Margarine	Margarines and spreads	Cheese, cream, margarine and butter substitutes	
Dressings (Mayonnaise, ketchup, dressings)	Meat and chicken extracts	Sauces, gravies and savoury spreads	
Salty snacks	Sweet or savoury packaged snacks	Packaged savoury snacks	
Crispbreads and rusks	Mass-produced packaged breads and buns	Industrial processed breads and buns	
	Package soup and noodles, Ready-to-heat/eat products and dishes (pies, pasta, pizza, desserts)	Ready-to-eat/heat food	Sausage rolls, Breaded fish Fast food meat and meat replacing products

Table S2. Descriptive characteristics in the individual cohorts of the HELIX project

Cohorts (N = 1138)	BIB	EDEN	INMA	KANC	MOBA	RHEA
N	201	146	185	196	211	199
Proportion of UPF (mean(SD)) (UPF ingested / total servings)	6.63 (0.24)	10.77 (0.54)	8.75 (0.54)	6.47 (0.49)	8.50 (0.50)	6.53 (0.28)
Child sex = male (%)	111 (55.2)	83 (56.8)	103 (55.7)	106 (54.1)	113 (53.6)	111 (55.8)
Child age (mean (SD)) (years)	6.63 (0.24)	10.77 (0.54)	8.75 (0.54)	6.47 (0.49)	8.50 (0.50)	6.53 (0.28)
Child white European ancestry = yes (%)	86 (42.8)	146 (100.0)	185 (100.0)	196 (100.0)	202 (95.7)	199 (100.0)
Child sedentary behaviour (mean (SD)) (min/day)	237.84 (135.79)	188.45 (81.19)	209.98 (82.98)	361.37 (164.61)	211.44 (116.52)	218.98 (91.47)
Child bmi (mean (SD)) (kg/m2)	15.95 (2.03)	18.00 (2.93)	17.91 (3.01)	16.41 (2.30)	16.37 (1.91)	16.77 (2.56)
Total vegetables intake (%) (servings/week)						
< 6	31 (15.4)	70 (47.9)	81 (43.8)	95 (48.5)	30 (14.2)	65 (32.7)
6.0 - 8.5	59 (29.4)	37 (25.3)	59 (31.9)	64 (32.7)	76 (36.0)	89 (44.7)
> 8.5	111 (55.2)	39 (26.7)	45 (24.3)	37 (18.9)	105 (49.8)	45 (22.6)
Total fruit intake (%) (servings/week)						
< 7	31 (15.4)	70 (47.9)	81 (43.8)	95 (48.5)	30 (14.2)	65 (32.7)
7 - 14.1	59 (29.4)	37 (25.3)	59 (31.9)	64 (32.7)	76 (36.0)	89 (44.7)
> 14.1	111 (55.2)	39 (26.7)	45 (24.3)	37 (18.9)	105 (49.8)	45 (22.6)
Maternal education (%)d						
Low	95 (47.3)	10 (6.8)	44 (23.8)	12 (6.1)	0 (0.0)	9 (4.5)
Medium	34 (16.9)	53 (36.3)	74 (40.0)	69 (35.2)	43 (20.4)	110 (55.3)
High	72 (35.8)	83 (56.8)	67 (36.2)	115(58.7)	168 (79.6)	80 (40.2)
maternal bmi (mean (SD)) (kg/m2)	28.17 (5.28)	23.29 (4.23)	23.79 (4.38)	27.60 (5.03)	22.71 (3.27)	24.09 (4.26)
maternal age (mean (SD)) (years)	28.64 (5.78)	30.77 (4.91)	31.85 (4.01)	29.12 (4.92)	32.73 (3.70)	30.86 (4.76)
maternal active smoking = yes (%)	11.11 (6.43)	13.48 (5.66)	13.08 (6.43)	15.63 (6.39)	13.73 (5.15)	14.44 (6.26)

Table S3. Quality Control EWAS individual cohorts

	# CpGs originally included	# CpGs excluded	# CpGs after exclusion	% CpGs excluded	Array type
HELIX	386518	13204	373314	3.4	450K
GXXI	764015	64259	699756	8.4	EPIC
ALSPAC	433793	11668	422125	2.7	450K
GenR	458563	44115	414448	9.6	450K

Table S4. Inflation factor lambda and 95% CI for each adjustment model, Fixed Effects Meta-analysis**.**

Model 1	Model 2	Model 3	Ancestry	Healthy diet
1.00<br>[1.03,0.96]	1.01<br>[1.05,0.98]	0.99<br>[1.02,0.96]	0.97<br>[1.00, 0.94]	0.98<br>[1.01,0.95]

Table S5 Random effect meta-analysis for the top CpGs associated with ultra-processed food intake in children.

Model	β	SE	p-value	Estimate direction (HELIX, G21, ALSPAC, GenR)	I^2
**cg14665028**					
Model 1	0.001	0.000	2.3E-04	+ + + +	11.1%
Model 2	0.001	0.000	1.8E-05	+ + + +	0.0%
Model 3	0.001	0.000	5.3E-06	+ + + +	0.0%
Ancestry	0.001	0.000	2.7E-05	+ + + +	0.0%
Healthy diet	0.001	0.000	1.3E-04	+ + + +	0.0%
**cg18968409**					
Model 1	0.006	0.001	4.5E-07	+ + + +	0.0%
Model 2	0.006	0.001	9.3E-07	+ + + +	0.0%
Model 3	0.006	0.001	9.4E-07	+ + + +	0.0%
Ancestry	0.006	0.001	3.6E-07	+ + + +	0.0%
Healthy diet	0.006	0.001	1.3E-06	+ + + +	0.0%
**cg00339913**					
Model 1	-0.003	0.001	9.7E-06	- - - -	0.0%
Model 2	-0.004	0.001	4.1E-06	- - - -	0.0%
Model 3	-0.004	0.001	7.0E-06	- - - -	0.0%
Ancestry	-0.003	0.001	3.0E-05	- - - -	0.0%
Healthy diet	-0.004	0.001	2.2E-06	- - - -	0.0%
**cg24730307**					
Model 1	0.001	0.000	2.3E-05	+ + + +	0.0%
Model 2	0.001	0.000	2.0E-05	+ + + +	0.0%
Model 3	0.001	0.000	4.8E-06	+ + + +	0.0%
Ancestry	0.001	0.000	6.2E-06	+ + + +	0.0%
Healthy diet	0.001	0.000	2.3E-05	+ + + +	0.0%
**cg03041696**					
Model 1	-0.002	0.000	3.8E-06	- - - -	4.9%
Model 2	-0.002	0.000	5.5E-06	- - - -	2.4%
Model 3	-0.002	0.000	2.0E-05	- - - -	8.7%
Ancestry	-0.002	0.000	2.6E-05	- - - -	6.8%
Healthy diet	-0.002	0.000	4.7E-06	- - - -	0.0%
**cg09709951**					
Model 1	0.005	0.001	3.5E-05	+ + + ?	0.0%
Model 2	0.005	0.001	2.8E-05	+ + + ?	0.0%
Model 3	0.006	0.001	1.2E-05	+ + + ?	0.0%
Ancestry	0.006	0.001	5.9E-06	+ + + ?	0.0%
Healthy diet	0.006	0.001	6.6E-06	+ + + ?	0.0%
**cg03999434**					
Model 1	-0.002	0.001	6.0E-03	- - - -	45.6%
Model 2	-0.002	0.001	1.2E-03	- - - -	37.2%
Model 3	-0.002	0.001	1.4E-03	- - - -	36.6%
Ancestry	-0.002	0.001	3.4E-04	- - - -	28.9%
Healthy diet	-0.002	0.001	1.8E-03	- - - -	34.0%

Model 1 includes basic potential confounders related to the child (ethnicity, age, and sex). Model 2 extends adjustments to maternal variables like smoking, education level, maternal BMI and age. Model 3 (main model) further includes child's sedentary behaviour and BMI. Two sensitive analysis models are reported: “Ethnicity” that only includes European children (n = 3007 samples), and “Healthy diet” further adjusting for fruit and vegetable consumption. Suggestive CpGs (p-value$<1^{-5}$) are in bold.

Table S6. Leave-one-out analysis: fixed effect meta-analysis of the 7 suggestive CpGs ($p<10^{-5}$), identified in Model 3 in meta-analysis across 4 cohorts

Model	β	SE	p.value	Estimate direction (HELIX, G21, ALSPAC, GenR)	I2
**cg14665028**					
Model 3 - all	0.0012	0.0003	5.3E-06	+ + + +	0.0%
Out Helix	0.0009	0.0004	9.8E-03	+ + +	0.0%
Out GXXI	0.0014	0.0003	2.8E-05	+ + +	0.0%
Out ALSPAC	0.0013	0.0003	1.3E-05	+ + +	0.0%
Out GenR	0.0012	0.0003	8.6E-06	+ + +	0.0%
**cg18968409**					0.0%
Model 3 - all	0.0057	0.0012	9.4E-07	+ + + +	0.0%
Out Helix	0.0058	0.0016	1.8E-04	+ + +	0.0%
Out GXXI	0.0059	0.0013	1.2E-05	+ + +	0.0%
Out ALSPAC	0.0054	0.0013	2.4E-05	+ + +	0.0%
Out GenR	0.0057	0.0012	3.9E-06	+ + +	0.0%
**cg00339913**					
Model 3 - all	-0.0036	0.0008	7.0E-06	- - - -	0.0%
Out Helix	-0.0032	0.0010	1.4E-03	- - -	0.0%
Out GXXI	-0.0041	0.0009	7.7E-06	- - -	0.0%
Out ALSPAC	-0.0034	0.0009	2.2E-04	- - -	0.0%
Out GenR	-0.0036	0.0009	3,3E-05	- - -	0.0%
**cg03041696**					
Model 3 - all	-0.0016	0.0004	3.3E-06	- - - -	8.7%
Out Helix	-0.0013	0.0005	1.3E-02	- - -	20.6%
Out GXXI	-0.0019	0.0004	8.1E-07	- - -	0.0%
Out ALSPAC	-0.0017	0.0004	2.7E-05	- - -	39.0%
Out GenR	-0.0016	0.0004	2.7E-05	- - -	25.3%
**cg24730307**					
Model 3 - all	0.0012	0.0003	4.8E-06	+ + + +	0.0%
Out Helix	0.0011	0.0004	2.5E-03	+ + +	0.0%
Out GXXI	0.0012	0.0003	3.9E-05	+ + +	0.0%
Out ALSPAC	0.0012	0.0003	2.3E-05	+ + +	0.0%
Out GenR	0.0012	0.0003	1.4E-05	+ + +	0.0%
**cg03999434**					
Model 3 - all	-0.0017	0.0004	2.2E-05	- - - -	36.6%
Out Helix	-0.0020	0.0006	3.6E-04	- - -	49.5%
Out GXXI	-0.0018	0.0004	3.9E-05	- - -	52.2%
Out ALSPAC	-0.0012	0.0004	5.1E-03	- - -	0.0%
Out GenR	-0.0017	0.0004	2.2E-05	- - -	54.8%

(*) Only those CpGs that are present in 75% of the studies are meta-analysed (therefore, cg09709951 not present in GenR not included

Table S7. List of studies and CpGs reported to be associated with diet (diet quality, nutrient or food items)

Study	Exposure	Population	Cutoff	CpGs
**Domínguez-Barragán J, et al.** Blood DNA methylation signature of diet quality and association with cardiometabolic traits. *Eur J Prev Cardiol*. 2024 31(2):191-202. doi: 10.1093/eurjpc/zwad317.	Diet quality (DASH, Healthy-plant based diet, or Mediterranean Diet score)	Adults (whole blood)	146 CpGs (FDR < 0.05)	cg00826902,cg01947769,cg02923485,cg03541887,cg04031093,cg05304729, cg05399785,cg05603985,cg07035242,cg09823288,cg11832534,cg12728588, cg12736206,cg16395997,cg24254842,cg26227957,cg00939727,cg01648237, cg04232816,cg13354241,cg15357118,cg19790321,cg24230340,cg25296103, cg00686926,cg01896761,cg03084350,cg04523589,cg12381416,cg12992827, cg15131784,cg19375583,cg25851277,cg00741986,cg03358154,cg14459011, cg21121843,cg02107842,cg02739870,cg04605590,cg05575921,cg06298346, cg12884551,cg18447299,cg18491039,cg18520851,cg20691612,cg22472360, cg27128984,cg01702055,cg04305673,cg04424621,cg05400196,cg05689413, cg08956463,cg09016348,cg09387914,cg12269535,cg18305324,cg25114611, cg27395200,cg01676795,cg04816311,cg04907244,cg07559427,cg07571951, cg08774868,cg10401362,cg15571623,cg16567172,cg20592700,cg20641531, cg22407942,cg23261443,cg04137490,cg04445427,cg13518625,cg14930065, cg16847396,cg22524061,cg23900905,cg25073708,cg03290131,cg06700877, cg11849692,cg17485681,cg21377950,cg23761815,cg26955383,cg00574958, cg02079413,cg13059136,cg15945333,cg20045320,cg27305772,cg00508575, cg03074946,cg06647068,cg08640498,cg11185549,cg19788934,cg22488164, cg25113008,cg01543583,cg02003183,cg06633543,cg13976502,cg23919111, cg00881300,cg02119938,cg05438378,cg24125648,cg24263283,cg25790365, cg01678580,cg03699074,cg03746015,cg03819286,cg04995976,cg09018739, cg10922280,cg00639656,cg02650017,cg02909097,cg18061543,cg18181703, cg20761853,cg24240870,cg00711496,cg00994936,cg02455723,cg07458272, cg07573872,cg08363114,cg16158874,cg16655795,cg26470501,cg27160284, cg02744249,cg04306926,cg16691087,cg25046878,cg24564491,cg03318904, cg15770575
**Lecorguillé M, et al.** Association between dietary patterns reflecting one-carbon metabolism nutrients intake before pregnancy and placental DNA methylation. *Epigenetics*. 2022;17(7):715-730. doi: 10.1080/15592294.2021.1957575.	Dietary patterns (‘varied and balanced, vegetarian tendency, and ‘bread and starchy food’ , micronutrient intake)	Newborns (placenta)	DMRs with <=2 CpGs sites with a p-value < 0.05	DMR-Chr3: cg10769891, cg19132762, cg23658326, cg11600697, cg21490561, cg00893636, cg03192963, cg06791151
				DMR-Chr19: cg10755961, cg24874111, cg18587364, cg20631204, cg14073063, cg14308647
				DMR-Chr11: cg02831587, cg26240185, cg03326059, cg10572969, cg24412501
				DMR-Chr10: cg16989281, cg00086670, cg25143771, cg08856941, cg24592962
				DMR-Chr8: cg22012530, cg01134012, cg18198896, cg14721632, cg23090207, cg12686110, cg15541193, cg09317036
				DMR-Chr5: cg13505794, cg13911629, cg27366305

**Küpers LK, et al.** Maternal Mediterranean diet in pregnancy and newborn DNA methylation: a meta-analysis in the PACE Consortium. *Epigenetics*. 2022. 17(11):1419-1431. doi: 10.1080/15592294.2022.2038412.	rMED and/or rMEDp	Newborn (cord blood)	23 CpGs ($P < 1 \times 10^{-5}$)	cg14773728 cg11946165 cg20239381 cg24007300 cg21217540 cg11994984 cg23757341 cg09738156 cg20348703 cg26053358 cg11894854 cg13477178 cg23757341 cg13477178 cg11894854 cg11747820 cg09738156 cg20348703 cg07203767 cg26053358 cg11529346 cg11747820 cg26664457
**Ma J, et al.** Whole Blood DNA Methylation Signatures of Diet Are Associated With Cardiovascular Disease Risk Factors and All-Cause Mortality. *Circ Genom Precis Med*. 2020;13(4):e002766. doi: 10.1161/CIRCGEN.119.002766	MDS	Adults (whole blood)	14 CpGs FDR <0.05 ($P=1.5\times10^{-6}$)	cg06126421, cg05575921, cg16969872, cg03646329, cg02716826, cg04885881, cg02079413, cg19693031, cg25189904, cg12075928, cg02097604, cg08732950, cg18181703, cg01940273
	AHEI		24 CpGs FDR <0.05 ($P=6\times10^{-6}$)	cg09940677, cg24694018, cg02097604, cg25909064, cg26470501, cg19202384, cg11468085, cg02508743, cg031909891, cg11250194, cg07805029, cg27118035, cg20842915, cg16969781, cg03646329, cg27039118, cg24735226, cg05232694, cg18181703,cg16936953, cg08884571, cg25953130,cg13074055,cg01294327
**Karabegović I, et al.** Epigenome-wide association meta-analysis of DNA methylation with coffee and tea consumption. *Nat Commun*. 2021;12(1):2830. doi: 10.1038/s41467-021-22752-6.	Coffee consumption	Adults (peripheral blood)	11 CpGs ($P < 1.1 \times 10^{-7}$)	cg05575921, cg25648203, cg03636183, cg21161138, cg15928106, cg11550064, cg09935388, cg20228731, cg06126421, cg14476101, cg23916896
	Tea consumption		2 cpgs ($P < 5.0 \times 10^{-6}$)	cg20099906, cg05804170
**Lai CQ, et al.** Carbohydrate and fat intake associated with risk of metabolic diseases through epigenetics of CPT1A. *Am J Clin Nutr*. 2020 11;112(5):1200-1211. doi: 10.1093/ajcn/nqaa233.	Carbohydrate and fat intake		1 cpg	cg00574958
**Ott R, et al.** Epigenome-Wide Meta-analysis Reveals Associations Between Dietary Glycemic Index and Glycemic Load and DNA Methylation in Children and Adolescents of Different Body Sizes. *Diabetes Care*. 2023 Nov 1;46(11):2067-2075	Dietary GI	Children and adolescents (blood)	P nominal	cg0000807, cg01578632, cg11616283,cg15042047,cg08077269
	Dietary GL		P nominal	cg20274553,cg02978505,cg20965602,cg22542699,cg05855306
**Lecorguillé M, et al.** Maternal and Paternal Dietary Quality and Dietary Inflammation Associations with Offspring DNA Methylation and Epigenetic Biomarkers of Aging in the Lifeways Cross-Generation Study. *J Nutr*. 2023 Apr;153(4):1075-1088. doi: 10.1016/j.tjnut.2023.01.028.	Maternal E-DII	Children	$P < 1x10^{-5}$	cg20748132, cg00109781, cg13993877, cg26871350, cg26381263, cg22070649, cg06708956, cg01488575, cg14336308, cg24284539
	Maternal HEI			cg21840035, cg15478184, cg04776779, cg01455766, cg06199676, cg22082469, cg05437285, cg00109781, cg11468003, cg04839673
	Maternal DASH			cg15119693, cg10210739, cg08661219, cg20095560, cg20552468, cg17859359, cg25364619, cg22806934, cg17746360, cg18045100
	Paternal E-DII			cg16918683, cg22431767, cg26790423, cg20916830, cg08287737, cg07879720, cg16898495, cg24285545, cg13400365, cg13374264
	Paternal HEI-2015			cg22431767, cg15311954, cg18506400, cg14977608, cg20135776, cg20595323, cg08955721, cg14833293, cg03271761, cg25618378

Table S8. List of diet-related CpGs replicated in our study with a nominal p value (p < 0.05)

lit_cpg	Study and Cutoff	Cutoff	*Exposure*	Lit_beta	Beta_UPF	Pval_UPF
cg24240870 (*GOSR1*)	Domínguez-Barragán J, et al, 2024	FDR < 5%	*Diet quality*	0.049	0.002	0.001
cg05689413 (*MED20*)	Domínguez-Barragán J, et al, 2024	FDR < 5%	*Diet quality*	0.050	0.002	0.004
cg01896761 (*FBLN2*)	Domínguez-Barragán J, et al, 2024	FDR < 5%	*Diet quality*	-0.048	0.002	0.004
cg02978505 (intergenic)	Ott R, et al. 2023	P nominal	*Dietary GL*	-0.066	0.002	0.004
cg12728588 (*NCDN*)	Domínguez-Barragán J, et al, 2024	$P<1.08x10^{-7}$	*Diet quality*	-0.057	0.002	0.004
cg15928106 (*FLJ43663*)	Karabegović I, et al, 2021	$P < 1.1 \times 10^{-7}$	*Coffee*	0.002	0.002	0.005
cg13059136 (*SNORA54;NAP1L4*)	Domínguez-Barragán J, et al, 2024	FDR < 5%	*Diet quality*	-0.039	-0.002	0.006
cg08774868 (*SEMA3C*)	Domínguez-Barragán J, et al, 2024	$P<1.08x10^{-7}$	*Diet quality*	-0.063	-0.001	0.009
cg09940677 (*CDC42BPB*)	Ma J, et al. 2020	FDR <0.05	*Diet quality*	−0.001	0.002	0.009
cg16395997 (*WDR8*)	Domínguez-Barragán J, et al, 2024	FDR < 5%	*Diet quality*	-0.044	-0.003	0.010
cg09935388 (*GFI1*)	Karabegovic et al, 2021	P < 1.1 × 10−7.	*Coffee*	−0.001	-0.003	0.010
cg19132762 (*EPM2AIP1;MLH1*)	Lecorguillé M, et al. 2022	DMRs CpGs p-value < 0.05	*Dietary pattern*	0.000	0.001	0.017
cg07203767 (intergenic)	Küpers LK, et al, 2022	P < 1 × 10−5	*Maternal Med diet*	0.088	-0.001	0.017
cg25648203 (*AHRR*)	Karabegović I, et al, 2021	P 1.1 × 10−7.	*Coffee*	−0.001	-0.001	0.022
cg18520851 (*CDO1*)	Domínguez-Barragán J, et al, 2024	FDR < 5%	*Diet quality*	-0.054	0.002	0.025
cg20641531 (*ICA1*)	Domínguez-Barragán J, et al, 2024	FDR < 5%	*Diet quality*	-0.042	-0.002	0.047

(*) In bold the replicated diet-realted CpGs with an opposite direction to UPF-related CpGs

Table S9. Suggestive CpGs (p-value<10^{-4}) associated with UPF, and list of CpGs and genes used in enrichment analyses (Model 3)

CpGId	beta	se	p.value	Estimate direction*	i2	Gene Name	chr
cg18968409	0.0057	0.0012	9.38E-07	+ + + +	0.00		2
cg03041696	-0.0016	0.0004	3.32E-06	- - - -	0.09		14
cg24730307	0.0012	0.0003	4.77E-06	+ + + +	0.00		22
cg14665028	0.0012	0.0003	5.33E-06	+ + + +	0.00	NHEJ1	2
cg00339913	-0.0036	0.0008	6.95E-06	- - - -	0.00	PHYHIP	8
cg09709951	0.0059	0.0013	1.24E-05	+ + + ?	0.00	ATF7	12
cg03999434	-0.0017	0.0004	2.19E-05	- - - -	0.37		12
cg10908625	0.0003	0.0001	2.56E-05	+ ? + +	0.00	RAP2B	3
cg26295786	0.0025	0.0006	2.95E-05	+ + + -	0.06		14
cg25208215	0.0004	0.0001	3.25E-05	+ + + +	0.00	C3orf57	3
cg14844466	-0.0020	0.0005	3.81E-05	- - - -	0.00	CNIH	14
cg02537382	0.0035	0.0009	4.19E-05	? + + +	0.19		20
cg04929612	0.0027	0.0007	4.73E-05	+ + + +	0.44		8
cg08999272	-0.0013	0.0003	4.89E-05	- - + -	0.00	RPTOR	17
cg08380539	0.0005	0.0001	5.42E-05	+ + + +	0.00	CTBS	1
cg18395558	0.0005	0.0001	5.54E-05	+ + + +	0.48	RTBDN	19
cg02317763	0.0003	0.0001	5.93E-05	+ + + +	0.00	GFOD2	16
cg01788360	0.0006	0.0002	5.95E-05	+ + + +	0.00	SRD5A3	4
cg16586594	-0.0067	0.0017	6.02E-05	- - - -	0.00	ABAT	16
cg18640098	0.0019	0.0005	6.13E-05	+ + + +	0.00	LOC285692	5
cg12999296	0.0003	0.0001	6.37E-05	+ + + +	0.17		13
cg17445155	-0.0036	0.0009	6.41E-05	- - - -	0.48	PPEF2	4
cg19363970	0.0014	0.0004	9.04E-05	+ + + +	0.00	TBC1D4	13
cg07845873	-0.0029	0.0007	9.06E-05	- - - -	0.25		3
cg14264125	0.0029	0.0007	9.40E-05	+ + + +	0.21		7
cg10935138	-0.0005	0.0001	9.70E-05	? - - -	0.00	WBP2	17
cg04508371	-0.0008	0.0002	9.98E-05	- - - -	0.00		1

*Estimate direction studies order is (HELIX, G21, ALSPAC, GenR)

Table S10. Top 10 of enriched pathways from GO and KEGG gene sets with a nominal p value ($p < 0.05$)

ID[a]	ONTOLOGY[b]	TERM	N[c]	DE[d]	P.DE	FDR
		GO				
GO:0051338	BP	regulation of transferase activity	857	6	0.001	1
GO:0008134	MF	transcription factor binding	565	5	0.001	1
GO:0010799	BP	regulation of peptidyl-threonine phosphorylation	43	2	0.002	1
GO:0102389	MF	polyprenol reductase activity	1	1	0.002	1
GO:0016095	BP	polyprenol catabolic process	3	1	0.002	1
GO:1902444	MF	riboflavin binding	1	1	0.002	1
GO:0003867	MF	4-aminobutyrate transaminase activity	1	1	0.002	1
GO:0032144	CC	4-aminobutyrate transaminase complex	1	1	0.002	1
GO:0032145	MF	succinate-semialdehyde dehydrogenase binding	1	1	0.002	1
GO:0034386	MF	4-aminobutyrate:2-oxoglutarate transaminase activity	1	1	0.002	1
GO:0047298	MF	(S)-3-amino-2-methylpropionate transaminase activity	1	1	0.002	1
		KEGG				
hsa00010		Thyroid hormone signaling pathway	117	2	0.014	1
hsa00020		Non-homologous end-joining	13	1	0.018	1
hsa00030		Butanoate metabolism	24	1	0.029	1
hsa00040		Transcriptional misregulation in cancer	180	2	0.029	1
hsa00051		MicroRNAs in cancer	287	2	0.033	1
hsa00052		Steroid hormone biosynthesis	54	1	0.035	1
hsa00053		Maturity onset diabetes of the young	26	1	0.038	1
hsa00061		Propanoate metabolism	32	1	0.040	1
hsa00062		beta-Alanine metabolism	31	1	0.043	1
hsa00071		Autophagy - other	29	1	0.044	1
hsa00100		Alanine, aspartate and glutamate metabolism	36	1	0.047	1

[a] Gene Ontology (GO) or KEGG ID, a unique digit identifier prefixed by GO and KEGG

[b] Abbreviations: BP (Biological Process), MF (Molecular Function), or CC (Cellular Component).

[c] Number of genes of UPF-related CpGs overlapping with the genes annotated to the GO term.

[d] Total number of genes in the GO term.

Table S11. Top 10 Enriched tissue with a nominal p value < 0.05

Cell	Tissue	Datatype	Pvalue	Qvalue	Probe
E071 Brain Hippocampus Middle	Brain	TssAFlnk	0.0001	1	cg00339913, cg03041696, cg08380539, cg09709951, cg12999296, cg14844466, cg18968409, cg25208215
E129 Osteoblast Primary Cells	Bone	TssAFlnk	0.0023	1	cg00339913, cg03041696, cg07845873, cg08380539, cg09709951, cg14665028, cg14844466
E049 Mesenchymal Stem Cell Derived Chondrocyte Cultured	Mesenchymal	TssAFlnk	0.0023	1	cg00339913, cg03041696, cg03999434, cg07845873, cg08380539, cg09709951, cg14665028, cg14844466
E081 Fetal Brain Male	Brain	Het	0.0025	1	cg04929612, cg17445155, cg26295786
E055 Foreskin Fibroblast Primary Cells skin01	Epithelial	TssAFlnk	0.0031	1	cg00339913, cg03999434, cg07845873, cg09709951, cg14665028, cg14844466, cg25208215
E104 Right Atrium	Heart	Het	0.0058	1	cg04508371, cg18395558, cg18640098
E058 Foreskin Keratinocyte Primary Cells skin03	Epithelial	TssAFlnk	0.0065	1	cg00339913, cg03041696, cg07845873, cg09709951, cg14844466, cg17445155
E100 Psoas Muscle	Muscle	Het	0.0070	1	cg04508371, cg04929612
E065 Aorta	Heart	TssBiv	0.0076	1	cg03999434, cg10935138
E077 Duodenum Mucosa	Digestive	TssAFlnk	0.0081	1	cg00339913, cg03041696, cg07845873, cg09709951, cg14665028, cg14844466
E034 Primary T cells from peripheral blood	Blood	Het	0.0081	1	cg04508371, cg04929612
E031 Primary B cells from cord blood	Blood	EnhBiv	0.0083	1	cg03041696, cg03999434, cg25208215

Figure S1. Manhattan plot of the fixed effect meta-EWAS of UPF intake (Model 3), n=3152 children

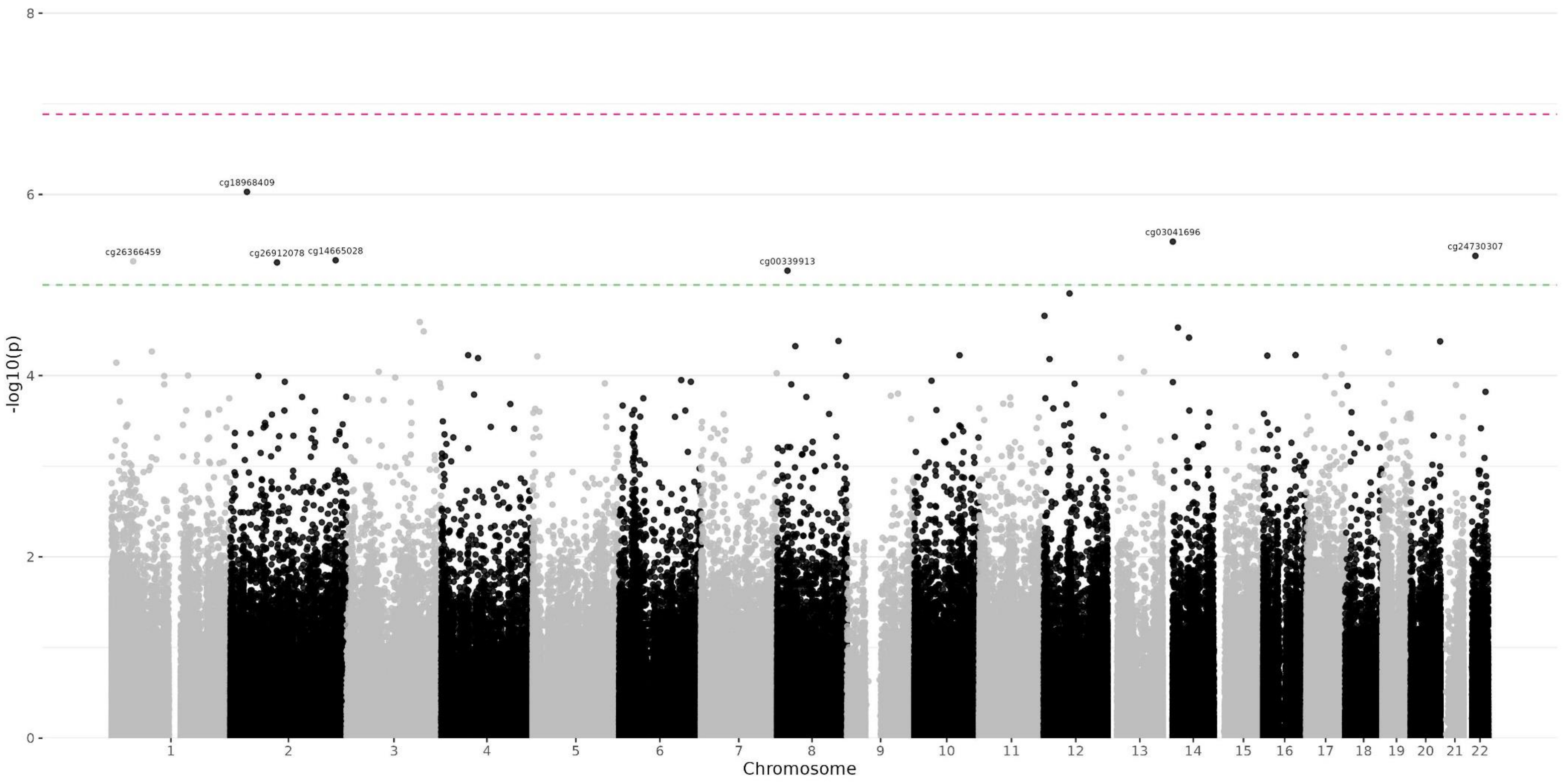

The plot represents the log-transformed p-values (y-axis) for the association between UPF intake and DNA methylation (DNAm), adjusted for ethnicity, age, sex, maternal factors (including smoking, education level, BMI, and age), as well as the child's sedentary behavior and BMI. CpG sites with a p-value less than 10^-5 are labeled. The red line indicates the Bonferroni threshold (p < 1e-08), and the green line represents the suggestive significance threshold (p < 1e-05).

Figure S2. Forest plots of the 7 suggestive CpGs associated with UPF intake across the 4 studies included in the meta-analysis (Model 3)

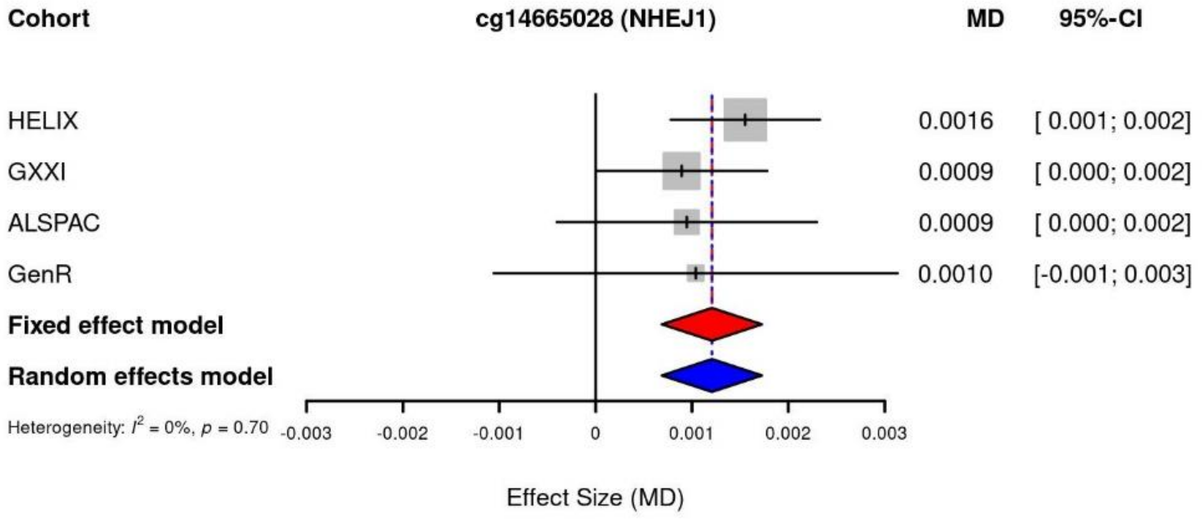

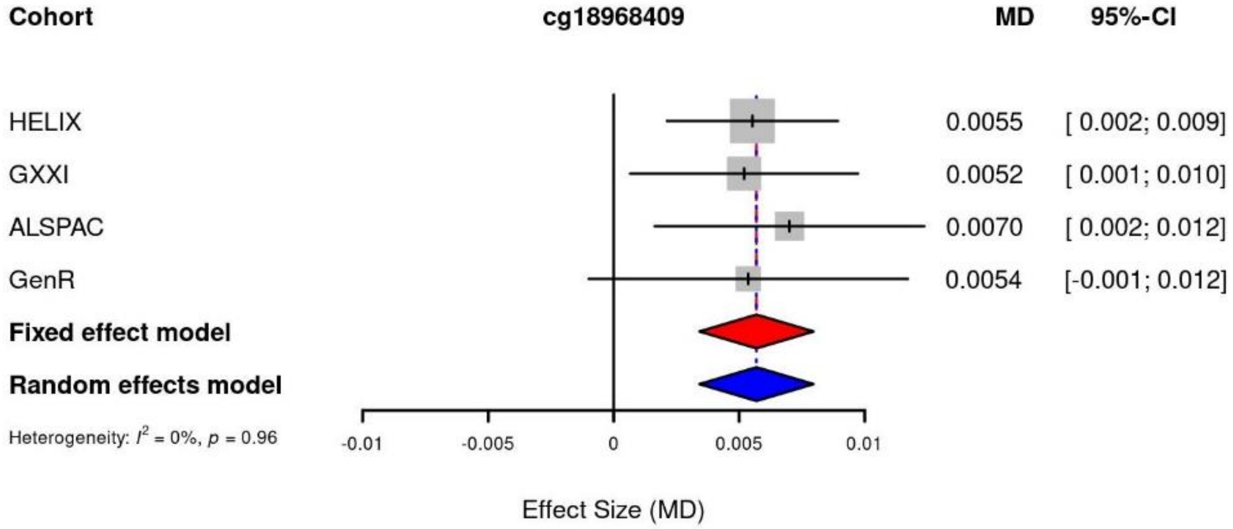

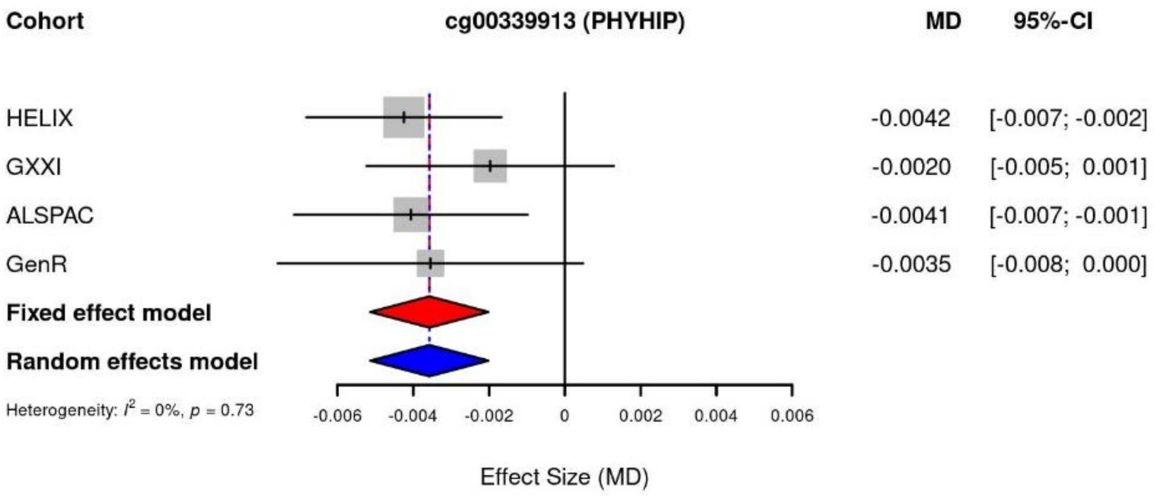
Cohort
cg00339913 (PHYHIP)
MD
95%-CI
HELIX
-0.0042
[-0.007; -0.002]
GXXI
-0.0020
[-0.005; 0.001]
ALSPAC
-0.0041
[-0.007; -0.001]
GenR
-0.0035
[-0.008; 0.000]
Fixed effect model
Random effects model
Heterogeneity: I^2 = 0%, p = 0.73
-0.006
-0.004
-0.002
0
0.002
0.004
0.006
Effect Size (MD)

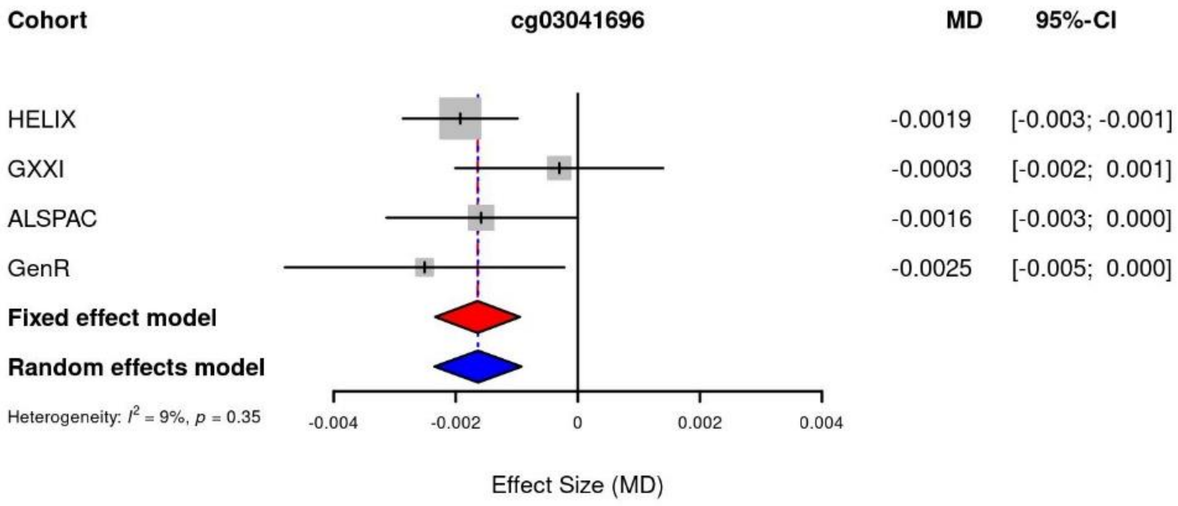
Cohort
cg03041696
MD
95%-CI
HELIX
-0.0019
[-0.003; -0.001]
GXXI
-0.0003
[-0.002; 0.001]
ALSPAC
-0.0016
[-0.003; 0.000]
GenR
-0.0025
[-0.005; 0.000]
Fixed effect model
Random effects model
Heterogeneity: I^2 = 9%, p = 0.35
-0.004
-0.002
0
0.002
0.004
Effect Size (MD)

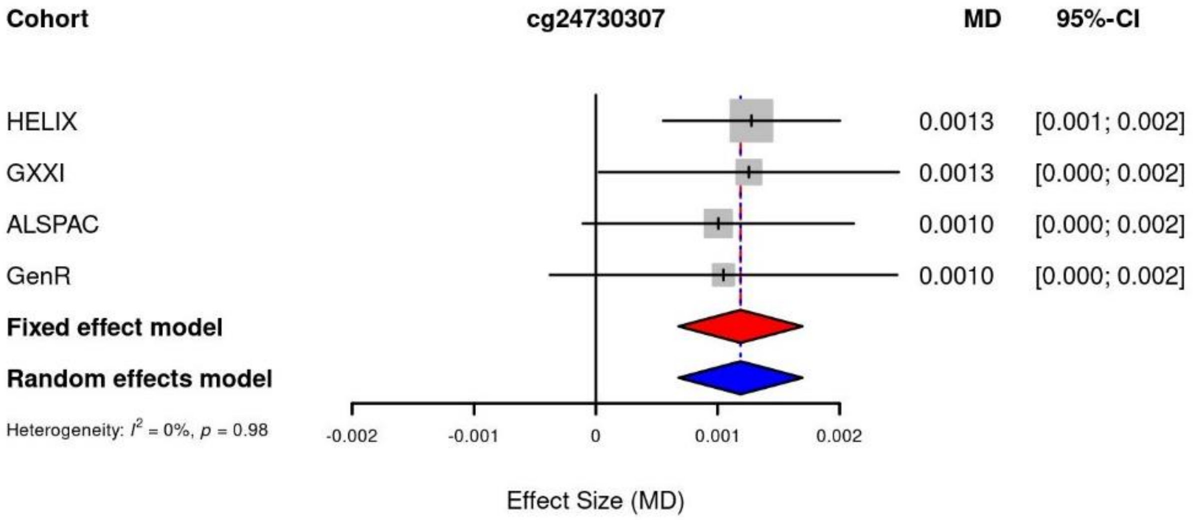

Cohort
cg24730307
MD
95%-CI
HELIX
0.0013
[0.001; 0.002]
GXXI
0.0013
[0.000; 0.002]
ALSPAC
0.0010
[0.000; 0.002]
GenR
0.0010
[0.000; 0.002]
Fixed effect model
Random effects model
Heterogeneity: I^2 = 0%, p = 0.98
-0.002
-0.001
0
0.001
0.002
Effect Size (MD)

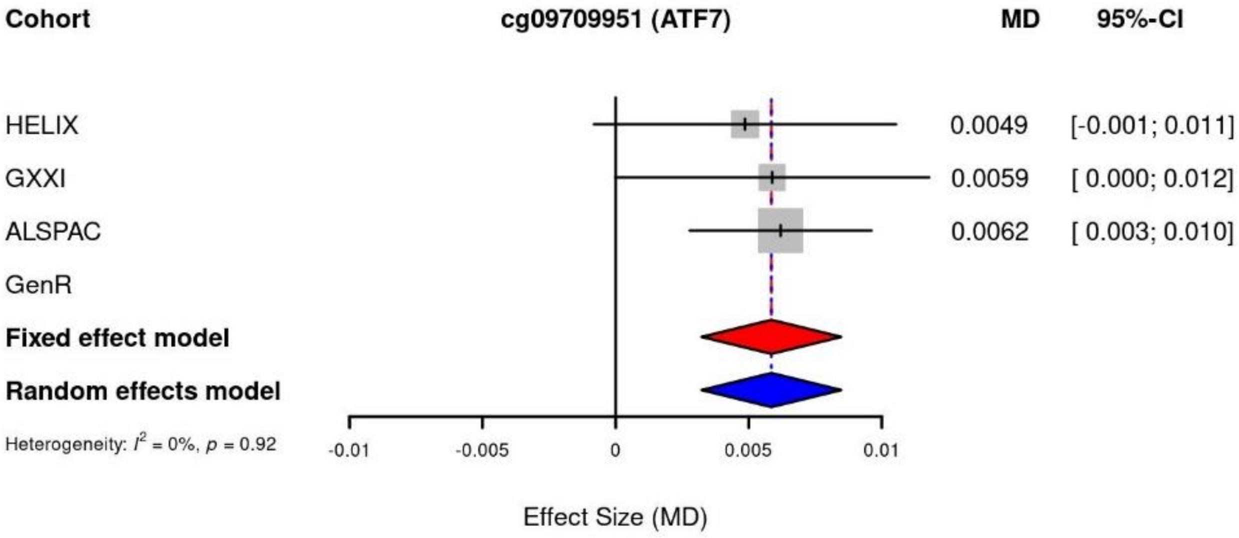

Cohort
cg09709951 (ATF7)
MD
95%-CI
HELIX
0.0049
[-0.001; 0.011]
GXXI
0.0059
[0.000; 0.012]
ALSPAC
0.0062
[0.003; 0.010]
GenR
Fixed effect model
Random effects model
Heterogeneity: I^2 = 0%, p = 0.92
-0.01
-0.005
0
0.005
0.01
Effect Size (MD)

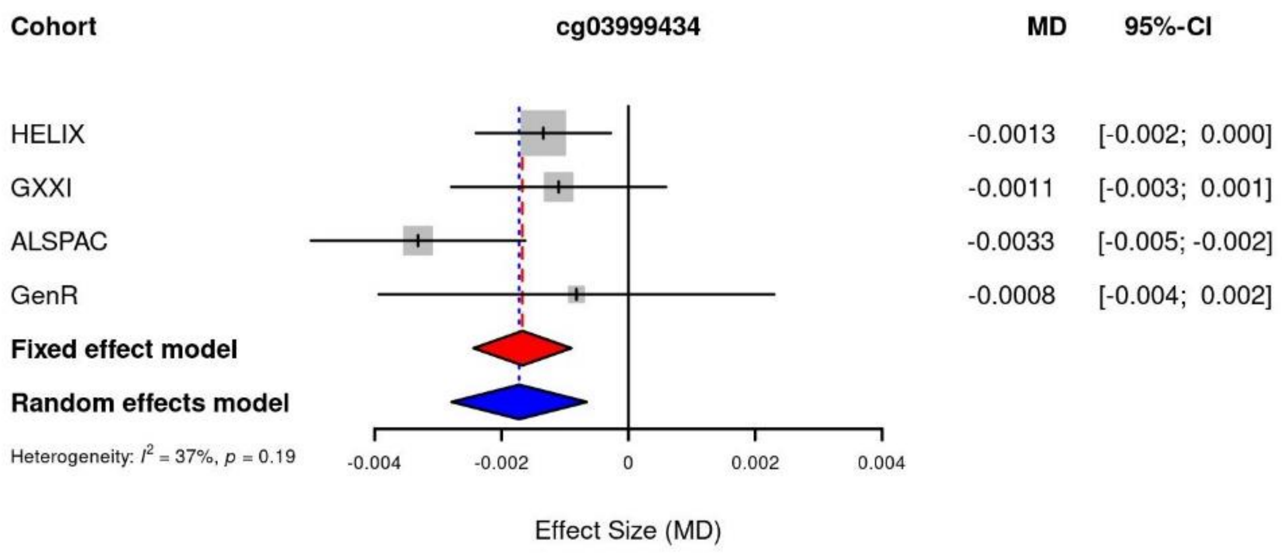
Cohort
cg03999434
MD
95%-CI
HELIX
-0.0013
[-0.002; 0.000]
GXXI
-0.0011
[-0.003; 0.001]
ALSPAC
-0.0033
[-0.005; -0.002]
GenR
-0.0008
[-0.004; 0.002]
Fixed effect model
Random effects model
Heterogeneity: I^2 = 37%, p = 0.19
-0.004
-0.002
0
0.002
0.004
Effect Size (MD)
